# Differential proteomic analysis highlights metabolic strategies associated with balhimycin production in *Amycolatopsis balhimycina *chemostat cultivations

**DOI:** 10.1186/1475-2859-9-95

**Published:** 2010-11-26

**Authors:** Giuseppe Gallo, Rosa Alduina, Giovanni Renzone, Jette Thykaer, Linda Bianco, Anna Eliasson-Lantz, Andrea Scaloni, Anna Maria Puglia

**Affiliations:** 1 Università di Palermo, Dipartimento di Biologia Cellulare e dello Sviluppo, Viale delle Scienze, Parco d'Orleans II, 90128 Palermo, Italy; 2 Proteomics & Mass Spectrometry Laboratory, ISPAAM, National Research Council, 80147 Naples, Italy; 3 Center for Microbial Biotechnology, Department of Systems Biology, Technical University of Denmark, Denmark

## Abstract

**Background:**

Proteomics was recently used to reveal enzymes whose expression is associated with the production of the glycopeptide antibiotic balhimycin in *Amycolatopsis balhimycina *batch cultivations. Combining chemostat fermentation technology, where cells proliferate with constant parameters in a highly reproducible steady-state, and differential proteomics, the relationships between physiological *status *and metabolic pathways during antibiotic producing and non-producing conditions could be highlighted.

**Results:**

Two minimal defined media, one with low Pi (0.6 mM; LP) and proficient glucose (12 g/l) concentrations and the other one with high Pi (1.8 mM) and limiting (6 g/l; LG) glucose concentrations, were developed to promote and repress antibiotic production, respectively, in *A. balhimycina *chemostat cultivations. Applying the same dilution rate (0.03 h^-1^), both LG and LP chemostat cultivations showed a stable steady-state where biomass production yield coefficients, calculated on glucose consumption, were 0.38 ± 0.02 and 0.33 ± 0.02 g/g (biomass dry weight/glucose), respectively. Notably, balhimycin was detected only in LP, where quantitative RT-PCR revealed upregulation of selected *bal *genes, devoted to balhimycin biosynthesis, and of *phoP*, *phoR*, *pstS *and *phoD*, known to be associated to Pi limitation stress response. 2D-Differential Gel Electrophoresis (DIGE) and protein identification, performed by mass spectrometry and computer-assisted 2 D reference-map http://www.unipa.it/ampuglia/Abal-proteome-maps matching, demonstrated a differential expression for proteins involved in many metabolic pathways or cellular processes, including central carbon and phosphate metabolism. Interestingly, proteins playing a key role in generation of primary metabolism intermediates and cofactors required for balhimycin biosynthesis were upregulated in LP. Finally, a bioinformatic approach showed PHO box-like regulatory elements in the upstream regions of nine differentially expressed genes, among which two were tested by electrophoresis mobility shift assays (EMSA).

**Conclusion:**

In the two chemostat conditions, used to generate biomass for proteomic analysis, mycelia grew with the same rate and with similar glucose-biomass conversion efficiencies. Global gene expression analysis revealed a differential metabolic adaptation, highlighting strategies for energetic supply and biosynthesis of metabolic intermediates required for biomass production and, in LP, for balhimycin biosynthesis. These data, confirming a relationship between primary metabolism and antibiotic production, could be used to increase antibiotic yield both by rational genetic engineering and fermentation processes improvement.

## Background

Glycopeptide antibiotics have found a successful use as last resort antibiotics in the treatment of methicillin-resistant *Staphylococcus aureus *(MRSA) infections [1]. Leading glycopeptide drugs are vancomycin and teicoplanin, produced by the actinomycetes *Amycolatopsis orientalis *and *Actinoplanes teichomyceticus*, respectively. The actinomycete *Amycolatopsis balhimycina *DSM5908, which produces the vancomycin-like antibiotic balhimycin [2,3], has been investigated as model strain for studies on glycopeptide biosynthesis since it can be genetically modified and the sequence of *bal *cluster, containing genes devoted to balhimycin biosynthesis, is available [4-6].

Balhimycin consists of a heptapeptide core made of the proteinogenic amino acids Leu and Asn and the nonproteinogenic amino acids 3,5-dihydroxyphenylglycine (DPG), 4-hydroxyphenylglycine (HPG) and β-hydroxytyrosine (H-Tyr). This heptapeptide is assembled by a non-ribosomal peptide synthetase (NRPS) and then extensively modified by the so called tailoring reactions, such as oxidative cross-linking of the electron-rich aromatic side chains, halogenation, glycosylation and methylation [6].

In Actinomycetes, the biosynthesis of secondary metabolites like antibiotics is generally elicited as developmental program and physiological response to a variety of environmental stimuli, including high cell density, nutritional limitation and/or presence of stress-inducing agents [7-9]. In *A. orientalis *[10,11] and *A. balhimycina *[12] cultivations, performed using minimal defined media, glucose amount was revealed positively correlated with antibiotic biosynthesis, cell growth rate and biomass production. On the other hand, inorganic phosphate (Pi) limitation is known to negatively affect growth rate and biomass production but to be beneficial for the production of glycopeptide antibiotics as shown for vancomycin [10], A40926 (produced by *Nonomuraea *ATCC 39727) [13] and balhimycin [12]. Pi limitation negatively controls the expression of both primary and secondary metabolism genes [14-16]. In Streptomycetes, Pi-controlled regulatory elements, called PHO boxes, have been reported in the upstream region of Pho regulon genes, which are devoted to Pi-nutritional stress response [15-19]. The PHO boxes are targets of PhoP, a transcriptional regulator whose activity is regulated by the phosphate-sensing membrane protein PhoR. PhoP is reported to have a dual role acting as either positive or negative regulator [16-20]. Although balhimycin biosynthesis has been extensively studied, the molecular bases of limitation of nutrients, such as Pi and glucose, controlling primary metabolism and antibiotic biosynthesis have not been investigated yet.

Proteomics was proven useful to identify specific biochemical pathways (or parts thereof) and key enzymes to be further targeted in genetic manipulations aimed to maximize the conversion of substrates into useful end-products in microorganisms [21,22]. In this context, we have recently used a differential proteomic approach to demonstrate that balhimycin production in batch culture is associated with the upregulation of enzymes involved in the biosynthesis of antibiotic precursors, thus suggesting that the metabolic apparatus is orientated to sustain balhimycin production [23,24]. Accordingly, an increased precursor availability, in particular tyrosine, was recently showed to be beneficial for balhimycin production [25].

Up to date, few experimental studies on antibiotic production have focused attention on continuous cultures, although the advantages of this type of study of microbial physiology have been recognized for many years. In fact, this approach provides useful means of researching the relationships between physiological *status *of an organism and production of antibiotics by comparing highly reproducible steady-state conditions, where growth parameters (i.e. biomass production yield, growth rate, carbon source uptake, O_2 _consumption, CO_2 _and metabolite production) and medium components are constant [10]. In this study, chemostat cultures of *A. balhimycina *were used to obtain steady-state conditions for biomass accumulation with the same growth rate and with or without balhimycin production. These cultivations were then analyzed by a comparative proteomic study to elucidate changes in the expression of genes involved in *A. balhimycina *primary and secondary metabolism which are associated with biomass production and antibiotic synthesis.

## Results and Discussion

### Chemostat cultivation profile

**Figure 1:**
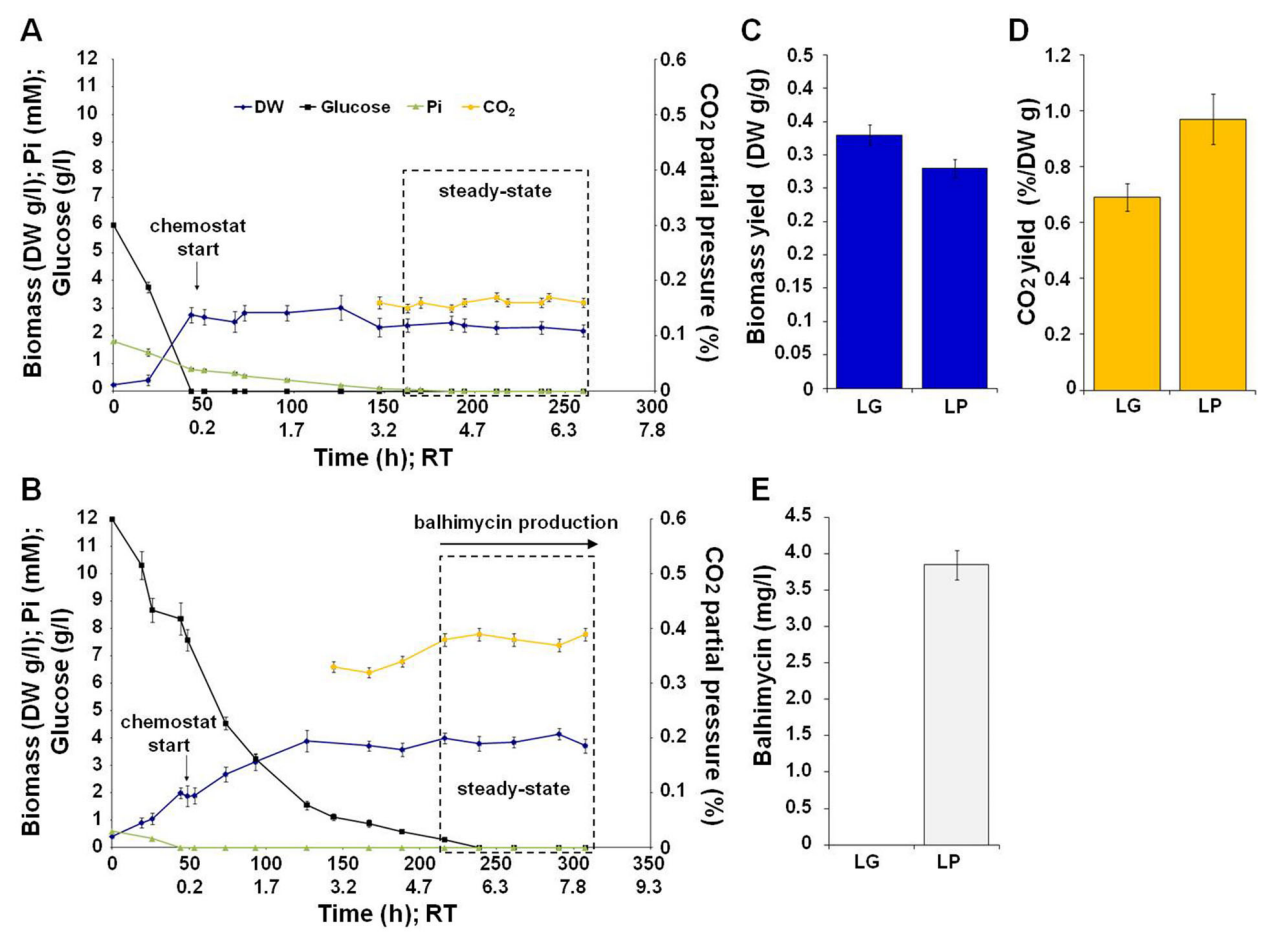
**Chemostat cultivation profile of *A. balhimycina *grown in LG (panel A) and LP (panel B) conditions**. The steady-state was reached after about 3 and 5 residence times (RT), respectively. RT, corresponding to 33.3 h, was calculated as the inverse of DR. Data shown per each condition are mean values generated by two parallel cultivations. Mean values during steady-state of: biomass dry weight (DW) production yield, normalized to the glucose consumption (panel C); CO_2 _production yield, reported as partial pressure (%) and normalized to the biomass DW concentration (panel D); balhimycin concentration (panel E). Vertical bars represent standard deviations.

Preliminary *A. balhimycina *batch cultivations, performed using a minimal defined medium containing glucose as only carbon source, revealed that to generate 1 g/l of biomass dry weight (BDW) about 3 g/l of glucose and 0.3 mM of Pi are necessary in a balanced growth. Thus, two minimal defined media, one with low Pi (0.6 mM; LP) and proficient glucose (12 g/l) concentrations and the other one with high Pi (1.8 mM) and limiting (6 g/l; LG) glucose concentrations, were developed to perform *A. balhimycina *chemostat cultivations. Appling a dilution rate of 0.03 h^-1 ^a highly reproducible and stable steady-state was achieved in both conditions (Figure 1: panels A and B), revealing that mycelia grew with the same rate and with a similar biomass production yield, calculated on consumed glucose, i.e. 0.38 ± 0.02 and 0.33 ± 0.02 g/g (BDW/glucose) in LG and LP, respectively (Figure 1, panel C). In these systems, residual Pi and glucose concentrations (Figure 1 panels A and B) were close to zero during steady-state, thus revealing that glucose and Pi, continuously introduced by feeding in, are completely up-taken during constant growth. Despite the similar glucose-biomass conversion coefficients, CO_2 _production yield, normalized to BDW, was about 1.4 fold higher in LP than in LG condition Figure 1 panel D). This result indicates an increased metabolism throughout TCA cycle in LP. Interestingly, as demonstrated by HPLC analysis, balhimycin was revealed only in LP condition, showing a productivity of 0.03 mg/g/h (balhimycin/BDW/time), and the antibiotic on-set coincided with CO_2 _production yield increment Figure 1 panel B). Conversely, LG condition resulted in a complete inhibition of antibiotic production. The biomasses, harvested during the steady-state, were then used to carry out gene expression analysis by quantitative (q)RT-PCR of selected genes and by differential proteomic analysis.

### Quantitative RT-PCR of Pho regulon genes

The expression of Pho regulon genes was used as reporter of Pi nutritional stress in chemostat cultures. In Streptomycetes, the products of these genes, like the phosphatase PhoD and the high affinity Pi transporter system PtsSABC, are known to provide extracellular Pi to fulfil the metabolic needs [15-19] or, like the polyphosphate kinase Ppk, to link energetic state of cell and Pi accumulation [26,27]. A BLAST analysis, performed using amino acid sequence of *S*. *coelicolor *PhoR, PhoP, PhoD, PstS and Ppk against *A. balhimycina *ORF database, revealed genes whose products show homology of 65%, 90%, 67%, 59% and 76% with their *S. coelicolor *counterparts, respectively. The identification of these *A. balhimycina *proteins was further confirmed by a BLAST analysis, performed using their amino acid sequence (Additional file 1 Table1S) against SwissProt database [28]. In fact, this analysis revealed high degree of homology (ranging from 85 to 100%) with actinomycete proteins annotated as PhoP, PhoR, PhoD, PstS and Ppk, respectively (Additional file 1 Table 2S).

**Figure 2:**
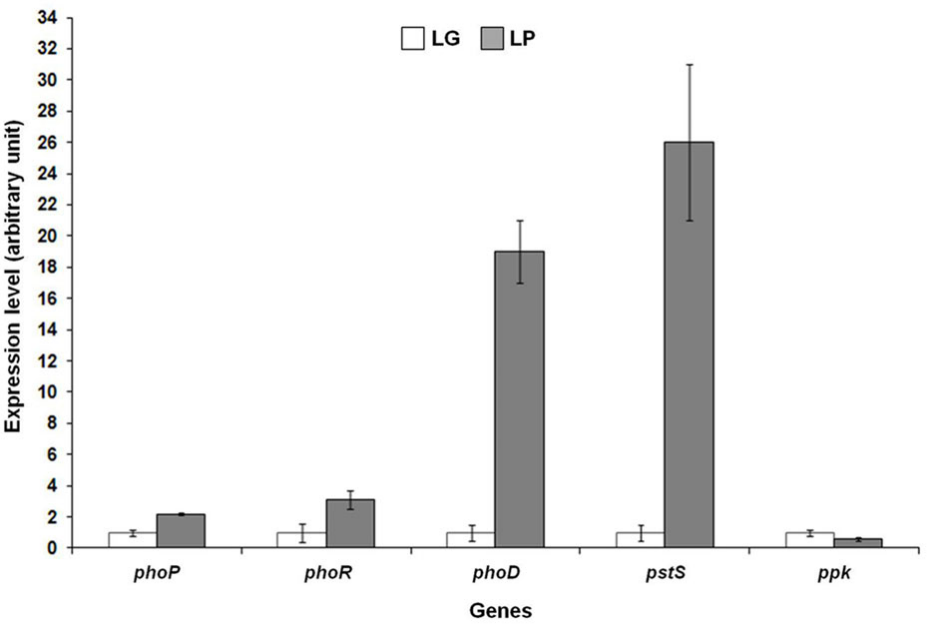
**Transcriptional expression profile of selected *A. balhimycina *Pho regulon genes**.

As revealed by qRT-PCR, all the tested genes but *ppk *were upregulated in LP. In particular, *phoR *and *phoP*, which also showed co-transcription (data not shown), were induced about 2-fold, while *pstS *and *phoD *more than 20-fold (Figure 2). In agreement with Rodríguez-García and co-workers (2007) that reported upregulation of *S*. *coelicolor *homologues [16] in phosphate-deficiency, these data revealed that Pi is limiting under LP condition.

Surprisingly, *ppk *expression was downregulated in LP (Figure 2). Ppk is reported to be involved in polyPi utilization in *S. lividans *[26] or in polyPi synthesis in *Bacillus cereus *[29] and *Myxococcus xanthus *[30]. The downregulation of *ppk *in LP condition suggests that in *A. balhimycina *the functional role of Ppk is to act mainly as polyPi kinase for Pi storing. In addition, accordingly to that it has already been observed in *S*. *lividan*s [27], the expression of *ppk *is negatively related to antibiotic production in *A. balhimycina*.

### Quantitative RT-PCR of *bal *genes

**Figure 3:**
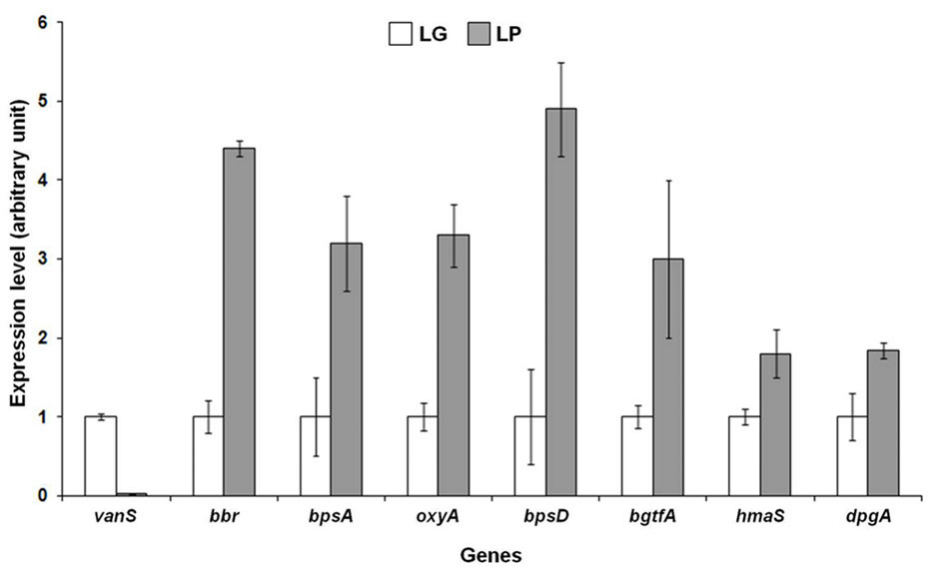
**Transcriptional expression profile of selected *A. balhimycina bal *genes**.

The expression of selected *bal *genes, essential for balhimycin production and chosen as representative of polycistronic transcripts [4], was analysed by qRT-PCR. The analysed *bal *genes are involved in DPG (*dpgA*), HPG (*hmaS*) and H-Tyr (*bpsD*) synthesis, in heptapeptide backbone assembling (*bpsA*) and tailoring reactions (*oxyA *and *bgtfA*), as well as in regulatory (*bbr*) and self-resistance (*vanS*) mechanisms. qRT-PCR revealed that all these genes are upregulated under LP condition (Figure 3), with the exception of *vanS*, thus revealing that the Pi limitation in glucose proficiency positively affects the expression of balhimycin biosynthetic genes. VanS is a membrane protein acting with the transcriptional regulator VanR as a two-component system putatively devoted to sense extracellular balhimycin. Its downregulation is in agreement with the mechanism proposed by Walsh *et al*. [31] where VanR negatively controls the expression of *vanSR *bicistronic transcript in the presence of glycopeptide antibiotics.

Under LG condition, the low expression levels of *bal *genes could not explain alone the absence of antibiotic production. In fact, this finding suggests complex regulatory mechanisms involving the synthesis of balhimycin precursors and the formation of the end-product. The importance of the availability of precursors in antibiotic biosynthesis will be elucidated by proteomic results.

### Proteomic analysis

**Figure 4:**
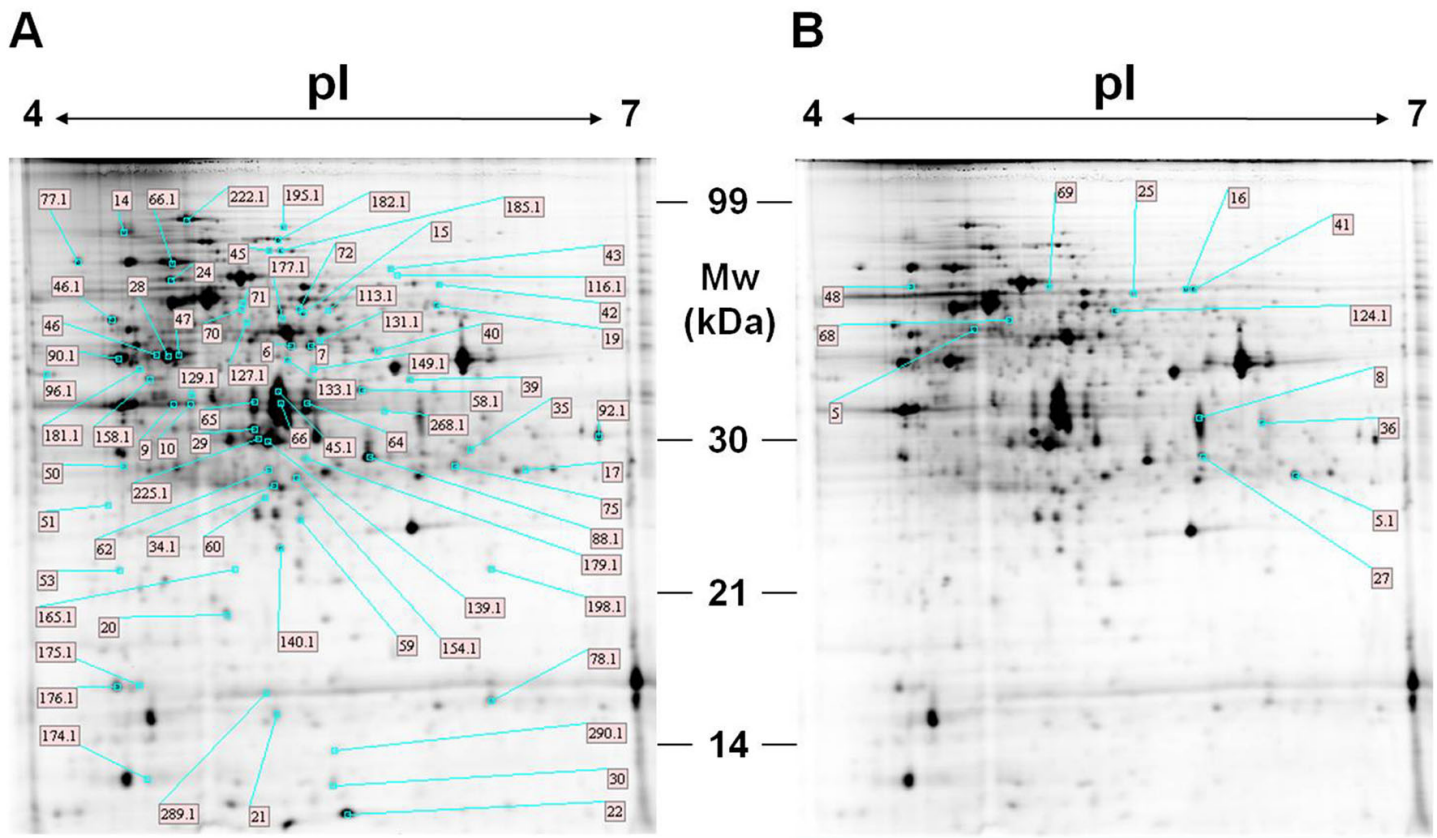
**2D-electropherograms, available over the World Wide Web as interactive pages at http://www.unipa.it/ampuglia/Abal-proteome-maps, of *A. balhimycina *protein extracts from biomass collected from LP (A) and LG (B) cultivations**. Labels indicate protein up-(A) or downregulated (B) in LP condition (Additional file 1 Table 3S).

Analysis of global protein expression changes between LP and LG conditions was performed by 2D**-**Differential Gel Electrophoresis (2D-DIGE) analysis (Figure 4). This investigation revealed 72 upregulated and 12 downregulated protein spots in LP condition, showing at least 1.5 fold increased or decreased abundance (calculated as spot Vol) and at least a probability for null hypothesis (p) < 0.05 (calculated using the analysis of variance or ANOVA test). These proteins were identified by MS analysis or by automatic gel-matching using the *A. balhimycina *protein 2 D reference-maps, available over the World Wide Web as interactive pages at http://www.unipa.it/ampuglia/Abal-proteome-maps[23,24] (Additional file 1 Table 3S, 4S, 5S and 6S). The reliability of this analysis was firstly demonstrated by the identification of groups of proteins showing similar expression profiles and whose corresponding genes are arranged in putative operons (Additional file 1 Table 6S), which are characterized by a maximum of 60 bp between two adjacent ORFs. The identified proteins were clustered into functional categories according to BioCyc http://biocyc.org/[32], KEGG http://www.genome.jp/kegg/[33] and ExPASy http://expasy.org/[28] metabolic databases. The functional clustering revealed that the differentially expressed proteins are related to many metabolic pathways or cellular processes, including glucose and phosphate metabolism. On the basis of their function, the identified proteins could be divided in several groups which are related to central carbon metabolism (31%), protein biosynthesis/amino acid metabolism (21%), redox and energetic balance (10%), nucleotide/fatty acid/amino sugar metabolism (10%) and balhimycin biosynthesis (2%) or are unknown (15%). The role of these proteins is discussed below in dedicated sections, highlighting the mechanisms controlling adaptation to Pi limitation, glucose catabolism and the metabolic strategies aimed to ensure metabolic intermediates for both biomass accumulation and antibiotic biosynthesis.

### Central carbon catabolism

**Figure 5:**
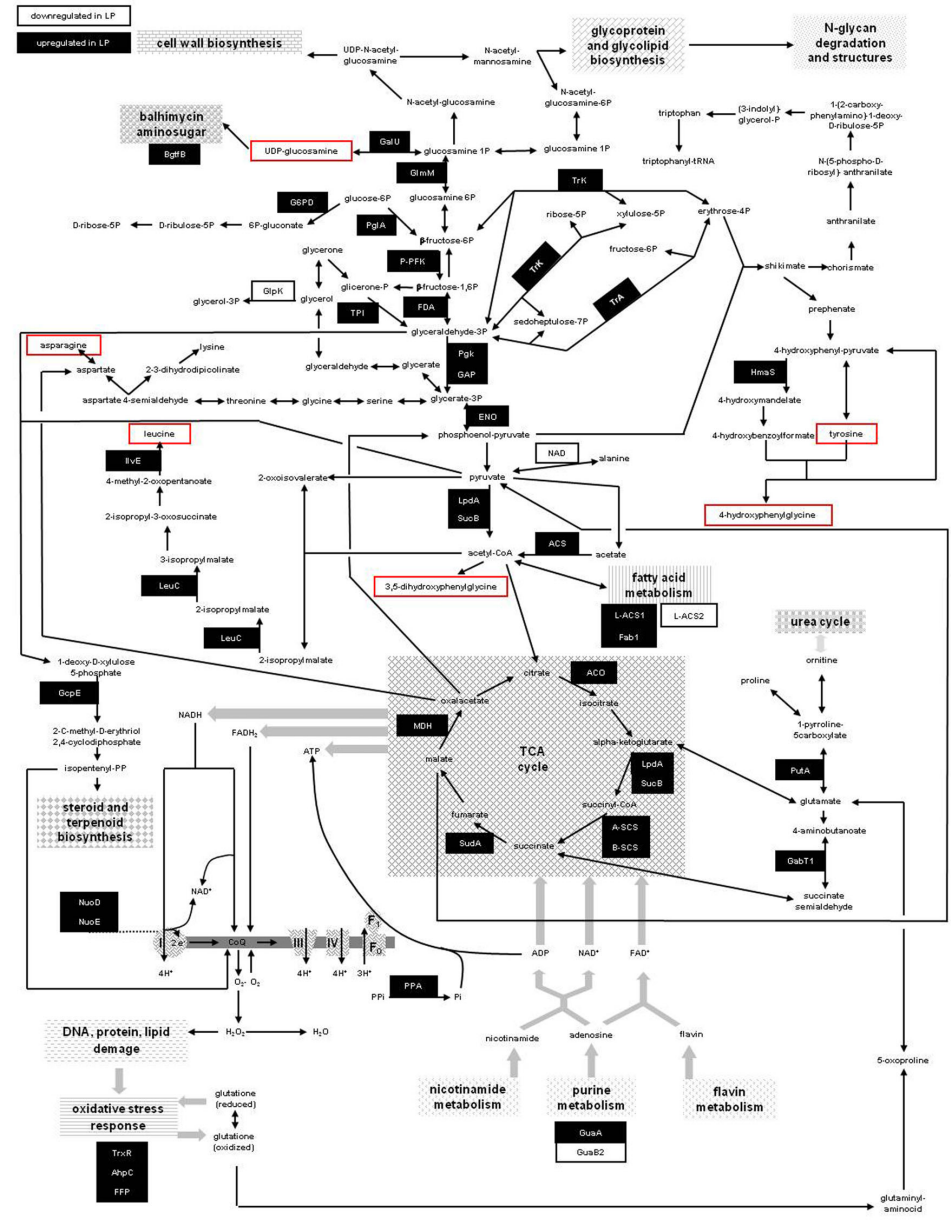
**Scheme of metabolic pathways where enzymes either upregulated (black boxes) or downregulated (white boxes) in LP are highlighted**. Upregulation of central carbon metabolism enzymes suggests an increased availability of primary metabolite intermediates and ATP, GTP, NAD(P)H and FADH_2 _for anabolic processes under LP condition. Enzymes related to the synthesis of balhimycin precursors (red rectangles), such as Leu, Tyr, Asn, DPG and HPG and amino sugars, were upregulated during antibiotic production. Reactions are reported according to KEGG and BioCy metabolic pathway databases. Enzyme name abbreviations refer to Table 3 S (Additional file 1).

Most differentially expressed proteins are involved in glycolysis, pentose phosphate pathway (PPP), and TCA cycle (Figure 5; Additional file 1 Table 3S). In particular, glycolytic enzymes glucose-6-phosphate isomerase (PgiA), triosephosphate isomerase (TPI), phosphoglycerate kinase (Pgk), glyceraldehyde 3-phosphate dehydrogenase (GAPDH), fructose-bisphosphate aldolase (FDA), PP-dependent-fructose 6-phosphate 1-phosphotransferase (P-PFK), enolase (ENO) and pyruvate dehydrogenase complex members (two dihydrolipoamide dehydrogenases, LpdA1 and 2, and one dihydrolipoamide acyltransferase, SucB) were upregulated in LP condition. The upregulation of these enzymes suggests an increased metabolic flux throughout glycolysis. This result is in agreement with the upregulation of the enzymes transketolase (TrK) and transaldolase (TrA), both belonging to non-oxidative branch of PPP, since these enzymes use as substrates the glycolytic intermediates fructose-6P and glyceraldehyde-3P, respectively. In addition, also the enzyme F420-dependent glucose-6-phosphate dehydrogenase (G6PD) was upregulated in LP, thus suggesting an increased flux throughout the oxidative branch of PPP as well. The upregulation of glycolytic enzymes is also in agreement with the upregulation of TCA cycle enzymes aconitate hydratase (ACO), succinate dehydrogenase flavoprotein subunit (SudA), LpdA1 and 2, SucB, succinyl-CoA ligase alpha and beta subunit (A-and B-SCS) and malate dehydrogenase (MDH) (Figure 5; Additional file 1 Table 3S). These findings also correlated with the increased production yield of CO_2 _in LP condition (Figure 1D) and also with the upregulation of ThiS and ThiC, involved in thiamine synthesis and previously associated with balhimycin production [23]. Thiamine diphosphate (TDP) is reported to be a co-factor of the upregulated TrK, pyruvate and alpha-ketoglutarate dehydrogenase complexes [34]. Finally, an ABC transport system ATPase component and an extracellular sugar-binding protein resulted downregulated in LP. These two proteins share conserved domains with LivG/F ATPases and MalE, respectively, which are shown to be required for the up-take of alternative carbon sources during glucose limitation/starvation [35,36].

The importance of glycolysis [37] and TCA cycle [10,16,38-40] for energetic balance and antibiotic production in Actinomycetes has already been reported. The upregulation of central carbon metabolism enzymes leads to an increased availability of NAD(P)H, FADH_2_, GTP and ATP and of primary metabolism intermediates. In fact, pyruvate and acetyl-CoA, from glycolysis, erytrose-4P, from PPP, and alpha-ketoacids from TCA cycle are required for anabolic routes such as amino acid biosynthesis (Figure 5). Interestingly, the upregulation of central carbon metabolism enzymes does not result in an increased biomass production yield, calculated on glucose consumption, in LP (Figure 1C). This result is most probably due to Pi limitation that negatively affects growth. On the contrary, the Pi proficiency in LG is counterbalanced by the low glucose availability that results in downregulation of central carbon metabolism enzymes. Thus, the similar biomass production yield in LP and LG conditions is achieved throughout a differential expression of central carbon metabolism key enzymes that provides for metabolic adaptation to energetic imbalance due to Pi limitation in LP condition and leads to an increased synthesis of primary metabolism intermediates and cofactors eventually required for biomass production and for balhimycin biosynthesis.

### Fatty acid metabolism

Two long-chain-fatty-acid-CoA ligases, namely L-ACS1 and 2, were upregulated and downregulated in LP, respectively (Figure 5; Additional file 1 Table 3S). Both are involved in the reversible formation of an acyl-carrier protein in the metabolism of fatty acids, with a mechanism leading to the release of PPi from ATP. In addition, the enzyme enoyl-ACP reductase (FabI), catalyzing the formation of trans-2,3-dehydroacyl-[acyl-carrier protein] and NADH from NAD^+ ^and enoyl-acyl-[acyl-carrier-protein] C4-16 derivatives, was upregulated in LP. This result is in agreement to Summers *et al*., (1999) [41] that revealed *fabI *being part of an operon induced in Pi limited cultures of *Sinorhizobium meliloti*. The downregulation of the glycerol kinase (GlpK) catalyzing glycerol phosphorylation, suggests a decreased synthesis of glycerolipids due to Pi limitation, which, instead, could be used as phosphorous source, as it has already been observed in *Bacillus subtilis *[42].

### Oxidative stress and hypothetical proteins

In agreement with the increased expression of TCA cycle enzymes, some upregulated proteins are involved in oxidative phosphorylation or counterbalance reactive oxidative species (ROS) generation and damage. In fact, upregulation was revealed for NADH:ubiquinone oxidoreductase proteins (NuoE and NuoD), 4-hydroxy-3-methylbut-2-en-1-yl diphosphate synthase (GcpE), involved in the synthesis of isopentenyl-PP that is a precursor of CoQ, AhpC, a thiol-specific antioxidant protein using reducing equivalents derived from either thioredoxin (TrxR) and glutathione, and TrxR, a small protein that alters the redox state of target proteins through the reversible oxidation of its active site (Figure 5; Additional file 1 Table 3S). These results are also in agreement with the increment of oxidative stress that has been already observed in *E coli *during Pi starvation [43]. In addition, upregulation of ferritin family protein (FFP) is in agreement with that it has already been observed in *S. coelicolor *[16] and in *S. meliloti *[44] and with a possible role in free iron detoxification for protection against ROS [45].

Hypothetical proteins (Additional file 1 Table 3S) that were previously associated with balhimycin production [23], such as a forkhead associated domain containing protein (FDCP) and a MoxR-domain containing ATPase, were also up-regulated in LP [46].

### Protein biosynthesis and nucleic acid metabolism

A number of proteins related to protein biosynthesis or protein folding and stress-damage response resulted upregulated in LP (Additional file 1 Table 3S). This is the case of elongation factor Tu (EF-Tu), trigger factor (TF), peptidyl-prolyl cis-trans isomerase (PPI), a cold shock-like protein (CSP-G), chaperone protein DnaK and Clp protease ATP-binding subunit (ClpB). In contrast, 60 kDa chaperonin GroEL and 30 S ribosomal protein S1 RpsA were downregulated under LP conditions (Additional file 1 Table 3S), as reported also in *S*. *coelicolor *[16], in *B. subtilis *[42] and in *S. meliloti *[44] incubated in Pi limiting condition. This result correlates with the chemostat cultivation conditions used, where mycelia are in a proliferating state and in LP a pool of upregulated factors, required for protein biosynthesis, may need to counterbalance energetic stress due to Pi limitation. In fact, the upregulation of factors necessary for either ribosomal activity or for protein folding together with the concomitant downregulation of the ribosomal protein RpsA, the largest ribosomal component with a pivotal role in ribosome assembly [47], may reflect the need to optimize the efficiency of protein biosynthesis under Pi nutritional stress conditions.

Under Pi limitation, bacteria may reduce their nucleoside pool [16] and accordingly inosine 5'-phosphate dehydrogenase (IMPDH), whose activity is required for the biosynthesis of xanthosine-5'P, a precursor of both guanosine and adenosine [48], was downregulated in LP. Surprisingly, guanosine 5'-phosphate (GMP) synthase, whose activity is associated with the formation of GMP and PPi from xantosine-5'P, was instead upregulated LP. This result might be explained since, as shown in KEGG pathway database, GMP synthase substrate (xanthosine-5'P) could derive from biochemical routes involving a pool of xantine and xantosine-triP or-tetraPi, in a salvage pathway that could be activated in Pi limitation.

### Balhimycin production

The amino acids H-Tyr, HPG, DPG, Asn and Leu are necessary to form the balhimycin heptapeptide backbone. Their availability from primary metabolism pathways is crucial for antibiotic biosynthesis [25]. Non-proteinogenic amino acids H-Tyr and HPG are also derived from metabolism of Tyr, which is also the amino donor during DPG biosynthesis. Genes encoding enzymes involved in the last steps of H-Tyr, HPG and DPG biosynthesis are present in the *bal *cluster, which also contain genes coding for enzymes required in the metabolism of amino sugars, essential for antibiotic glycosylation.

In agreement with balhimycin production observed only in LP, primary and secondary metabolism enzymes involved in the biosynthesis of balhimycin precursors, such as amino acids and amino sugars resulted upregulated in LP condition. In particular, the metabolic building blocks pyruvate, acetyl-CoA, oxalacetate, 4P-erythrose and glucose-6P from central carbon metabolism are required for Leu, DPG, Asn, Tyr, HPG and amino sugar biosynthesis, respectively (Figure 5) [25]. Thus, *bal *gene product HmaS-involved in HPG synthesis and whose expression was revealed upregulated by qRT-PCR-, 3-isopropylmalate dehydratase large subunit (LeuC) and branched-chain amino acid aminotransferase (IlvE)-catalyzing different reactions in Leu biosynthesis-, MDH-catalyzing biosynthesis of Asp-precursor oxalacetate-, *bal *gene product Bgtf, phosphoglucosamine mutase (GlmM) and UTP-glucose-1-phosphate uridylyltransferase (GalU)-required in the metabolism of amino sugars-were upregulated in LP. GlmM is particularly interesting because it catalyzes the formation of glucosamine-1P, a metabolic intermediate that is converted into UDP-glucosamine from GalU (Figure 5; Additional file 1 Table 3S). UDP-glucosamine is reported to be likely the primary metabolism building block required for the biosynthesis of modified amino sugars in glycopeptide teicoplanin, chloroeremomycin, and balhimycin [49]. Interestingly, a 4-phosphopantetheinyl transferase (PPT) was upregulated in LP. PPTs form a superfamily of enzymes that transfer prosthetic 4-phosphopantetheine moiety from CoA to carrier domain of biosynthetic complexes required for the synthesis of a wide range of compounds including fatty acid, polyketide (PK) and nonribosomal peptide (NRP) metabolites. As revealed by BLAST analysis, *A. balhimycina *PPT possesses conserved domains characteristic of Sfp-like phosphopantetheinyl transferases family. Members of this family are mainly found associated either with NRP synthetase and PK synthase [50]. Thus, even if an involvement in fatty acid biosynthesis could not be totally excluded, PPT might likely be involved in both balhimycin heptapeptide assembly line and in the synthesis of DPG, a polyketide derivative, synthesized by *dpgABCD *products [51].

Altogether, these data suggest that the destination of metabolic intermediates in LP and LG cultivations is differentially regulated. In particular, the increment of glucose catabolism in LP condition is coupled with the synthesis of metabolic building blocks related to the generation of both balhimycin backbone and amino sugar moieties.

### Identification of PHO box regulatory elements in the upstream of differentially expressed genes

**Figure 6:**
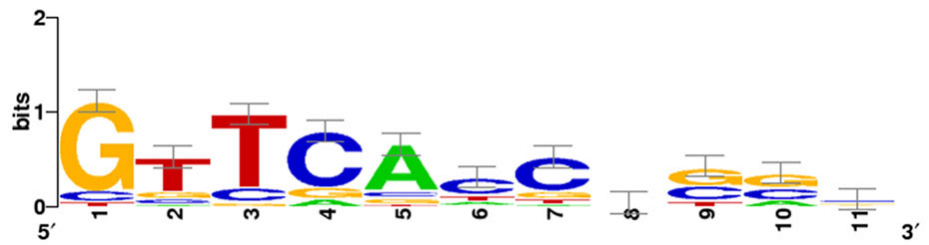
***Consensus *of the direct repeats of 11 nt that forms the *A. balhimycina *PHO box**. This logo corresponds to a model that comprises 19-DRs from nine *A. balhimycina *genes (Additional file 1 Tables 6S and 7S). The height of each letter is proportional to the frequency of the base; the height of the letter stack is the conservation in bits at that position. Error bars are shown at the top of the stacks.

Global gene expression analysis was proven as a good starting point to identify common regulatory elements, such as PHO boxes, in the upstream region of differentially expressed genes in *S. coelicolor *[16]. Similarly, putative PHO box regulatory elements, upstream the translational start site of differentially expressed genes, were searched by sequence homology using published *S. coelicolor *PHO box *consensus *[18]. Thus, PHO box directed repeats (DRs) in the upstream region of the genes encoding PstS, Ppk, PhoD, MDH, B-SCS, HmaS, Gabt1, LpdA2 and P-PFK were revealed by bioinformatics (Additional file 1 Table 7S and 8S). The *consensus *of *A*. *balhimycina *DRs (Figure 6), created using free-on line available WebLogo software http://weblogo.berkeley.edu/logo.cgi[52], shows high similarity with that of *S*. *coelicolor *revealing a high conservation of the first seven positions [18].

**Figure 7:**
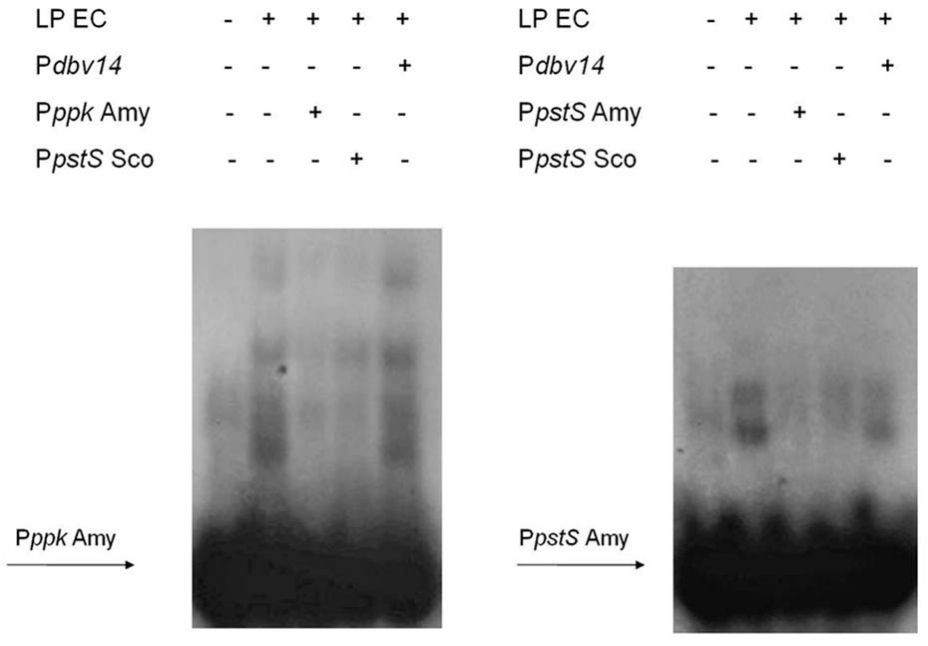
**Gel band shift assays**. EMSA was carried out using DNA fragments upstream *A. balhimycina ppk *(P*ppk *Amy) and *pstS *(P*pstS *Amy) and a crude extract from *A. balhimycina *LP culture. The use of a molar excess (200 fold) of unlabeled probe as well as of a DNA fragment upstream *S. coelicolor pstS *(P*pstS *Sco), containing a PHO box, reduced the band shift signals for both probes. No reduction of band shift signals is visible using a molar excess (200 fold) of an unspecific competitor (*Nonomuraea *P*dbv14 *[14]).

Experimental validation was carried out by electrophoresis mobility shift assay (EMSA) for the putative PHO boxes in the upstream of *pstS *and *ppk*, which were up-and downregulated in LP respectively, as revealed by qRT-PCR. In particular, 40 and 38 bp DNA fragments containing the upstream regions of *A. balhimycina pstS *(P*pstS *Amy) and *ppk *(P*ppk *Amy), respectively, and *A. balhimycina *crude extracts from biomass generated in LP condition were used. This analysis showed that both P*pstS *Amy and P*ppk *Amy specifically bind to the crude extract (Figure 7). Notably, a 40 bp DNA fragment containing the PHO box of *pstS *from *S. coelicolor *(P*pstS *Sco) specifically competed to *A. balhimycina *crude extract bound, resulting in reduction of band shift intensity for both P*pstS *Amy and P*ppk *Amy. This result, revealing an interspecific competition between *S. coelicolor *and *A. balhimycina *PHO boxes, suggests the binding of PhoP to these *A. balhimycina *regulatory DNA elements. The presence of a PHO box in the upstream region of both up-and downregulated genes is in agreement with PhoP dual role as positive or negative regulator [17-20]. Even if the expression of *ppk *is positively and indirectly controlled by PhoP in *S. lividans *TK24 [26], it is neither PhoP-or Pi-controlled in *S. coelicolor *[16]. Instead, in *A. balhimycina *the putative PHO box in the upstream region of *ppk *could be explained by a negative control of PhoP over *ppk *expression.

Thus, the presence of PHO box regulatory elements in the upstream of differentially expressed genes suggests a PhoP-mediated mechanism for the regulation of their expression. Anyway, further investigations, such as the construction of a *A*. *balhimycina *Δ*phoP *strains and EMSAs carried out with a purified *A. balhimycina *PhoP, should be performed to better clarify the role of PHO box-like regulatory elements in controlling the expression of *A. balhimycina *genes in Pi limitation.

## Conclusions

This is the first report describing a differential proteomic analysis for a glycopeptide producer strain incubated in continuous cultivations. Comparative analyses of global expression profiles in non-producing and producing conditions in batch cultivations may be affected by growth rate and changes of medium component concentrations. In the chemostat conditions which were set-up in this study to compare *A. balhimycina *proteomes in producing and non producing conditions, mycelia grew with the same rate and with similar glucose-biomass conversion coefficients.

Both transcriptional and proteomic analyses highlighted that in LP and LG conditions energy balance and generation of primary metabolism intermediates are mainly regulated by controlling the expression of central carbon metabolism enzymes and proteins, such as PstS, PhoD and an inorganic pyrophosphatase (PPA), whose activity is required for Pi recovering. The upregulation in LP of central carbon metabolism genes does not result in an increased biomass production yield on glucose consumption. Instead, the upregulation of catabolic and anabolic enzymes, coupled with the upregulation of *bal *genes, accounts for the supply of cofactors and precursors, such as amino acids and/or amino sugars, that otherwise could eventually become limiting for balhimycin biosynthesis.

Altogether these data correlate with the fact that in Actinomycetes secondary metabolites are generally synthesized through multiple intracellular reactions, which are further affected by cofactor balance and regulatory circuits at different levels of cellular metabolism [53-55]. Thus, altogether these data could be used to re-design fermentation strategies that are difficult to be intuitively identified and for approaching the prediction of new genetic targets from primary metabolism genes to be engineered for a rational construction of antibiotic high-yielding producer strains.

## Materials and methods

### Strain and culture condition

The *A. balhimycina *DSM5908 strain used in this work was a gift from Prof. Wohlleben, University of Tubingen, Germany. The strain was stored in 1-ml cryotubes at -80°C in 15% glycerol and 8 g/l tryptic soy broth (Difco, Detroit, Mich.), at a biomass dry weight of approximately 0.2 g/ml. For seed culture preparations, a 250-ml baffled Erlenmeyer flask containing 50 ml of tryptic soy broth (Difco) was inoculated with one glycerol stock vial and incubated with shaking (150 rpm) at 30°C. After 48 h of incubation, this seed culture was used for inoculation of the fermentors (5% v/v). The fermentations were performed in 1 l double-jacketed Applikon fermentors (Applikon, Shiedam, Netherlands) containing 1 l medium with an agitation rate of 500 rpm and aeration at 1 vvm (air volume/working volume/min). Dissolved O_2 _tension was monitored with an O_2 _electrode (Mettler Toledo, Greifensee, Switzerland); it did not fall below 50% saturation during any of the fermentations. The pH was kept at a value of 7.0 by addition of 1 M NaOH; temperature was maintained at 30°C. The two fermentation media, named LG and LP for low glucose and phosphate, respectively, contained the compounds in the concentration reported in Additional file 1 Table 9S. LP and LG chemostat experiments were performed in two parallel replicates, respectively, using a constant dilution rate (DR) of 0.03 h^-1^. It took about 3 and 5 residence times (RT, calculated as the inverse of DR) to achieve the stabilization of growth parameters (steady-state) in LG and LP cultivations which was confirmed by the constant off-gas and biomass concentration. The biomass samples used for total protein and RNA extractions were harvested during steady-state after about 6.7 and 7.9 RT from LG and LP cultivations, respectively, and immediately frozen in liquid N_2 _and then stored at -80°C.

### Analysis of biomass dry weight

For biomass concentration, sampling was performed manually in triplicate from each cultivation. The optical density was measured on a UV-vis spectrophotometer UV mini 1240 from Shimadzu at 600 nm. For dry weight measurements, 3 ml samples were filtered through a pre-weight 45 mm pore size Sartorius filter and washed with 0.9% NaCl (w/w). The filters (with content) were dried 20 min at 150W in a microwave oven and weighted after cooling.

### Analysis of glucose and phosphorus utilization

Glucose concentration in spent medium was measured by high performance liquid chromatography (HPLC) (Agilent Technologies, Palo Alto, CA) equipped with an AMINEX HPX-87 H column (Bio-Rad Laboratories, Hercules, CA) working at 60 1C. 5 mM H_2_SO_4 _was used as mobile phase with a flow rate of 0.6 ml min^-1^. Standard UV index detection at 210 nm was used for quantification.

Phosphorus concentration in spent medium was analyzed using a spectrophotometric assay kit (Inorganic phosphorus 80; Abx Diagnostics, Montpellier, France) in an automatic analyzer (Cobas Miras plus; Roche, Basel, Switzerland).

### Analysis of CO_2 _in exhaust gas

The partial pressure of CO_2 _in the exhaust gas from the bioreactors was measured using a gas analyzer (Industrial emission monitor type 1311; Brüel & Kjaer, Denmark).

### Analysis of balhimycin

Balhimycin titers in the cultivations were detected and quantified using liquid chromatography mass spectrometry (LCMS) as described elsewhere [25]. LC-DAD-ESI+-MS data were acquired on an Agilent 1100 HPLC system (Agilent Technologies, Waldbronn, Germany) equipped with a diode array detector scanner. The HPLC system was connected to an Agilent MSD Ion trap operated in positive electrospray full scan mode (m/z 100-1500) with 4 scans per second. The amount of balhimycin was quantified using UV detection at 195 and 280 nm and calculated using a standard curve. Separation was done on a Phenomenex Gemini C18 110A column (2 × 100 mm^2^, 3 μm) with a flow rate of 0.3 ml/min at 40°C using water (pH 10.5 with 10 mM ammonium formate) and methanol solution in the following gradient: t = 0 min, 10% methanol; t = 24 min, 100% methanol; t = 30 min, 10% methanol.

### Protein extraction and separation for 2D-Differential Gel Electrophoresis (2D-DIGE) analysis

Frozen biomass samples, collected from two parallel cultivations for each condition, were sonicated in non reducing conditions using experimental procedures as previously described [23]. After dialysis against distilled water, at 4°C, and acetone precipitation at -20°C, proteins were dissolved in the appropriate 2D-DIGE lysis buffer (30 mM Tris, 7 M urea, 2 M thiourea, 4% CHAPS).The four protein extracts (one for each replica *per *each condition) were analyzed in two technical replicates. In particular, two 40 μg protein aliquots from each extraction were minimally labelled using 320 pmol Cy3 and 320 pmol Cy5 fluorescent dyes (CyDye™DIGE minimal labelling kit, GE Healthcare, Uppsala, Sweden), respectively, to account for florescence bias. Labelling reactions were carried out in the dark on ice for 30 min. and quenched with 0.2 mM lysine, according to manufacturer instructions. In addition, four 40 μg protein aliquots from a standard pool, generated by combining an equal amount of all the four protein extracts, were minimally labelled with 320 pmol Cy2 fluorescent dye (CyDye™DIGE, GE Healthcare) according to manufacturer instructions. To perform 2D-DIGE analysis, 40 μg of LG and LP labelled proteins were mixed, combining Cy3 and Cy5 fluorescent dyes, with the addition of 40 μg of internal standard Cy2-labelled proteins, thus generating a total of four protein mixes.

For isoelectrofocusing (IEF), DeStreak rehydration solution (GE Healthcare) containing 0.5% (v/v) IPG buffer (GE Healthcare) and 1% (w/v) DTE (Sigma) was added to each mix up to 340 μl (final volume). IEF was performed as previously described [23] using 4-7 pH range 18 cm-IPG strips (GE Healthcare) in an Ettan IPGphor III apparatus (GE Healthcare). The focused proteins were then separated using 12% sodium dodecyl polyacrylamide gels (SDS-PAGE) at 10°C in a Hettan Dalt six (GE Healthcare), with a maximum setting of 40 μA and 110 V per gel.

The four 2D-Gels were scanned with a DIGE imager (GE Healthcare) to detect cyanin-labeled proteins according to manufacturer's instructions. Differential gel analysis was performed automatically using Image Master 2D Platinum 7.0 DIGE software (GE Healthcare), according to the manufacturer's instructions. Protein spots were detected automatically and manually verified. Individual spot abundance was automatically calculated from the quadruplicated 2D-Gels as mean spot volume (Vol, i.e. integration of optical density over spot area) and normalized to the Cy2-labeled internal pooled standard. Protein spots showing more than 1.5 fold change in Vol, with a statistically significant ANOVA value (p < 0.05), were considered differentially abundant and identified as described in the following section by either MS analysis or by gel-matching procedures using *A. balhimycina *protein 2D reference-maps previously obtained [23,24].

### MS analysis and protein identification

Protein spots were excised from the 2D-Gels, alkylated, digested with trypsin and identified as previously reported [23]. Peptide mixtures were desalted by μZipTipC18 (Millipore, MA) using 50% (v/v) acetonitrile/5% (v/v) formic acid as eluent before MALDI-TOF-MS and/or nLC-ESI-LIT-MS/MS analysis.

In the case of MALDI-TOF-MS experiments, peptide mixtures were loaded on the MALDI target, using the dried droplet technique and α-cyano-4-hydroxycinnamic acid as matrix, and analyzed using a Voyager-DE PRO mass spectrometer (Applied Biosystems, USA) operating in positive ion reflectron, with an acceleration voltage of 20 kV, a nitrogen laser (337 nm) and a laser repetition rate of 4 Hz [56]. The final mass spectra, measured over a mass range of 700-6000 Da and by averaging 400-800 laser shots, were elaborated using the DataExplorer 5.1 software (Applied Biosystems) and manually inspected to get the corresponding peak lists. Internal mass calibration was performed with peptides deriving from trypsin autoproteolysis.

Tryptic digests were eventually analyzed by nLC-ESI-LIT-MS/MS using a LTQ XL mass spectrometer (ThermoFisher, San Jose, CA) equipped with a Proxeon nanospray source connected to an Easy-nanoLC (Proxeon, Odense, Denmark) [57]. Peptide mixtures were separated on an Easy C_18 _column (10 × 0.075 mm, 3 μm) (Proxeon). Mobile phases were 0.1% (v/v) aqueous formic acid (solvent A) and 0.1% (v/v) formic acid in acetonitrile (solvent B), running at total flow rate of 300 nL/min. Linear gradient was initiated 20 min after sample loading; solvent B ramped from 5% to 35% over 45 min, from 35% to 60% over 10 min, and from 60% to 95% over 20 min. Spectra were acquired in the range m/z 400-2000. Acquisition was controlled by a data-dependent product ion scanning procedure over the three most abundant ions, enabling dynamic exclusion (repeat count 2 and exclusion duration 60 s); the mass isolation window and collision energy were set to *m/z *3 and 35%, respectively.

MASCOT search engine version 2.2.06 (Matrix Science, UK) was used to identify protein spots unambiguously from an updated NCBI nonredundant database also containing the *A. balhimycina *ORF product database based on *A. balhimycina *DSM5908 genome sequencing [23] by using MALDI-TOF-MS data, a mass tolerance value of 40-80 ppm, trypsin as proteolytic enzyme, a missed cleavages maximum value of 2 and Cys carbamidomethylation and Met oxidation as fixed and variable modification, respectively. Candidates with a MASCOT score > 83 (corresponding to p < 0.05 for a significant identification) were further evaluated by the comparison with their calculated mass and pI values, using the experimental values obtained from 2-DE.

Raw data files from nLC-ESI-LIT-MS/MS experiments were searched with SEQUEST (ThermoFisher Scientific, USA) within the Proteome Discoverer software package (Thermo Fisher Scientific, San Jose, CA, USA, version 1.0 SP1) against the NCBI nonredundant database implemented by the preliminary version of the *A*. *balhimycina *DSM5908 ORF product database mentioned above [23]. Database searching was performed by selecting Cys carbamidomethylation as a fixed modification and Met oxidation as variable modification. Searches were carried out by using a mass tolerance value of 2.0 Da for precursor ion and 0.8 Da for MS/MS fragments, trypsin as proteolytic enzyme, a missed cleavages maximum value of 2. Other SEQUEST parameters were kept as default. Candidates with more than 2 assigned peptides with an individual SEQUEST score versus charge state > 1.5 for charged state (CS) 1, > 2.0 for CS 2, > 2.4 for CS 3, > 3.3 for CS 4, > 4.2 for CS 5, > 4.5 for CS 6 were considered confidently identified. Definitive peptide assignment was always associated to manual spectral visualization and verification.

The sequence of the identified proteins and homology analysis, performed using on-line available versions of BLAST search against UniProt Knowledgebase http://www.expasy.ch/tools/blast, are reported in Additional file 1 Table 3S and 4S.

Protein identification by gel-matching was automatically performed using *A*.* balhimycina *protein 2D reference-maps [23], available over the World Wide Web as interactive pages at http://www.unipa.it/ampuglia/Abal-proteome-maps[24], and Image Master 2D platinum 7.0 software. More than 50 highly reproducible protein spots were used as landmarks to perform automatic gel-matching that was then verified by an accurate visual inspection using 3 D view tool.

### Total RNA isolation, RT-PCR and qRT-PCR analysis

Gene expression analysis at the transcriptional level of selected Pho regulon and *bal *genes was performed according to Gallo *et al*. 2010 [23]. The mycelia were resuspended in 1 ml P-buffer [58] containing lysozyme (1 mg/ml) and RNase inhibitor RNaseOUT (Invitrogen) (40 U/ml), and then incubated for 10 min, at 37°C. The RNA was extracted by using the RNeasy midi kit (Qiagen) according to the manufacturer's instructions. DNase I (Roche) treatment was performed at 37°C, for 1 h, and ethanol precipitation in the presence of 0.1 vol of 3 M sodium acetate allowed recovery of the DNase-treated total RNA. After a washing step with 70% (v/v) ethanol and air drying, the RNA pellet was resuspended in water with RNase inhibitor RNaseOUT (200 U/ml). As control of RNA quality, a RT-PCR with 0.1 μg of total RNA and primer pairs internal to *hrdB *was carried out using the Superscript One-Step RT-PCR kit (Invitrogen) and the conditions indicated by the supplier. PCRs were performed on 0.5 μg of RNA samples using 40 cycles prior to exclude the presence of genomic DNA.

Primer pairs amplifying intragenic regions of the genes analysed by qRT-PCR are listed in Table 10S (Additional file 1). The identity of RT-PCR products was confirmed by sequencing. The high-capacity cDNA archive kit (Applied Biosystems) was used to retrotranscribe 2 μg of total RNA, extracted from LP and LG cultures, in a 100 μl of water (final volume). Gene expression was analyzed quantitatively by using Applied Biosystems 7300 real-time PCR system (Applied Biosystems). The expression of *hrdB*[23] was used as an internal control to quantify the relative expression of target genes. 2 μl of cDNAs were mixed with 12.5 μl of SYBR green PCR master mix (Applied Biosystem) and 10 pmol of each primer in a final volume of 25 μl. The PCR was performed under the following conditions: 2 min at 50°C and 10 min at 95°C, followed by 40 cycles of 15 s at 95°C and 1 min at 60°C. A dissociation reaction was eventually performed using a temperature gradient from 55 to 99°C by increasing 1°C/min. This procedure permitted recording the melting curve of the PCR products and, consequently, their specificity to be determined. A negative control (distilled water) was included in all real-time PCR assays, and each experiment was performed in triplicate or quadruplicate.

### Search for putative PHO box sequences

*A. balhimycina *PHO box sequences were searched by matching two directs repeats (**gttcacccggc **and **gttcatttacg**) of *S. coelicolor *PHO box in the upstream region of *pstS *gene with regions extending 300 bp upstream of the putative translation start sites of differentially expressed *A. balhimycina *genes. To this aim, on-line available versions of ClustalW http://www.ebi.ac.uk/Tools/clustalw2/index.html[59] and EMBOSS GUI matcher http://bips.u-strasbg.fr/EMBOSS/[60] and BLAST bl2seq http://blast.ncbi.nlm.nih.gov/Blast.cgi[61] software were used. Only outputs derived from all the three approaches were accepted (Additional file 1 Table 6S and 7S).

### Preparation of labeled DNA fragments

DNA fragments containing the upstream regions of *S. coelicolor pstS *(P*pstS*: tccacaggg**gttcacccggcgttcatttac**gcccttcggc) and the *A. balhimycina pstS *(P*pstS*: gaaaggctt**gttcactttgcgttcatctgga**caggggaac) and *ppk *(P*ppk*: ggtatcgcgttgagct**gttcatctgaccttcaccacgg**) were prepared by incubation of the corresponding oligonucleotides at 90°C for 10 min, followed by slow cooling to room temperature. The annealed products were recovered from nondenaturing 20% polyacrylamide gels by the crush-soak method [62] and labeled with T4 polynucleotide kinase (Invitrogen) according to the supplier's protocol.

### Preparation of *A. balhimycina *crude extract

*A. balhimycina *pellet was washed twice with crack buffer (10 mM Tris-HCl, pH 8.0, 0.5 mM EDTA, 0.3 mM DTT), resuspended in 5 ml of crack buffer, and disrupted by sonication. The cell debris was removed by centrifugation at 13,000 × *g *(20 min, 4°C), and the supernatant was stored at −80°C.

### Gel mobility shift assay

The gel mobility shift assay was performed according to Alduina *et al*., (2007) [14]. For the binding assay, *A. balhimycina *crude extract was dialyzed against distilled water over night at 4°C and, then, approximately 200 μg of proteins were incubated in 20 μl of 12.5 mM Tris-HCl (pH 7.5), 10% glycerol, 62.5 mM KCl, 0.75 mM DTT, and 5 mM MgCl_2_, containing 100 μg of poly(dI-dC)-poly(dI-dC) ml^-1^, for 10 min, at 4°C. After 15 min of incubation with 0.4 ng of ^32^P-labeled DNA, complexes and free DNA were resolved on non-denaturing 5% polyacrylamide gels run in 0.5× Tris-borate-EDTA buffer at 150 V for approximately 2 h [54] and then equilibrated in 10% acetic acid, dried, and subjected to autoradiography. For testing the specificity of binding, either unlabeled probe or a competitor DNA fragment containing the upstream region of *Nonomuraea *sp ATCC 39727 *dbv*14 [14] were added before incubation of the proteins and probe.

## Abbreviations

BDW: biomass dry weight. DPG: 3,5-dihydroxyphenylglycine. HPG: 4-hydroxyphenylglycine. H-Tyr: β-hydroxytyrosine. LG: limiting glucose and high phosphate. LP: low phosphate and proficient glucose. MS: mass spectrometry. Pi: inorganic phosphate.

## Declaration of competing interests

The authors declare that they have no competing interests.

## Authors' contributions

GG carried out chemostat cultivations, DIGE analysis, protein identification by gel-matching, real-time RT-PCR and EMSA experiments, BLAST analyses and wrote the draft manuscript. RA helped to perform real-time RT-PCR and EMSA experiments and to wrote the draft manuscript. GR carried out protein MS-identification. JT designed chemostat experiments, helped to perform chemostat cultivations and revised the manuscript. LB helped to perform protein MS-identification. AEL supervised chemostat experiments and revised the manuscript. AS supervised protein MS-identification and revised the manuscript. AMP conceived and supervised the study and participated in its design and coordination and revised the manuscript.

All authors read and approved the final manuscript.

## Supplementary Material

Additional file 1 **.pdf contains 10 tables reporting: **- amino acid sequence of *A. balhimycina *Pho regulon gene products (Table 1S);-BLAST analysis of PHO regulon gene products against SwissProt database (Table 2S);-list of differential expressed proteins with information about their relative function, relative expression value, either theoretical and measured values for molecular weight (Mw) and isoelectric point (pI), protein identification method (Table 3S);-amino acid sequence of the MS-identified *A. balhimycina *proteins (Table 4S);-BLAST analysis data, obtained by using UniProt databank of proteins identified by MS analysis (Table 5S);-list of *A. balhimycina *DSM5908 genes arranged in putative operons (Table 6S);-sequence of upstream regions of selected *A. balhimycina *genes, showing PHO box directed repeats (DR) identified by ClustalW and BLAST bl2seq analysis performed by using *S. coelicolor *PHO box DR in the upstream regions of *pstS *(Table 7S);-EMBOSS-GUI Matcher analysis of *A. balhimycina *PHO box DR sequence performed by using *S. coelicolor *PHO box DR in the upstream regions of *pstS *(Table 8S);-composition of fermentation media (Table 9S);-list of primers used for qRT-PCR experiments (Table 10S). Click here for file
