# Supplementary material for: Differential proteomic analysis highlights metabolic strategies associated with balhimycin production in Amycolatopsis balhimycina chemostat cultivations
Source: Microb Cell Fact. 2010 Nov 26;9:95. doi: 10.1186/1475-2859-9-95 (PMC3004843; doi:10.1186/1475-2859-9-95)
Supplement: Additional file 1 — .pdf contains 10 tables reporting: - amino acid sequence of A. balhimycina Pho regulon gene products (Table 1S);-BLAST analysis of PHO regulon gene products against SwissProt database (Table 2S);-list of differential expressed proteins with information about their relative function, relative expression value, either theoretical and measured values for molecular weight (Mw) and isoelectric point (pI), protein identification method (Table 3S);-amino acid sequence of the MS-identified A. balhimycina proteins (Table 4S);-BLAST analysis data, obtained by using UniProt databank of proteins identified by MS analysis (Table 5S);-list of A. balhimycina DSM5908 genes arranged in putative operons (Table 6S);-sequence of upstream regions of selected A. balhimycina genes, showing PHO box directed repeats (DR) identified by ClustalW and BLAST bl2seq analysis performed by using S. coelicolor PHO box DR in the upstream regions of pstS (Table 7S);-EMBOSS-GUI Matcher analysis of A. balhimycina PHO box DR sequence performed by using S. coelicolor PHO box DR in the upstream regions of pstS (Table 8S);-composition of fermentation media (Table 9S);-list of primers used for qRT-PCR experiments (Table 10S). [file 1475-2859-9-95-S1.PDF]

Differential proteomic analysis highlights metabolic strategies associated with balhimycin production in *Amycolatopsis balhimycina* chemostat cultivations

Giuseppe Gallo[a], Rosa Alduina[a], Giovanni Renzone[b], Jette Thykaer[c], Linda Bianco[b], Anna Eliasson-Lantz[c], Andrea Scaloni[b] and Anna Maria Puglia[a].

[a]*Università di Palermo, Dipartimento di Biologia Cellulare e dello Sviluppo, Viale delle Scienze, Parco d'Orleans II, 90128 Palermo, Italy.*

[b]*Proteomics & Mass Spectrometry Laboratory, ISPAAM, National Research Council, 80147 Naples, Italy.*

[c]*Center for Microbial Biotechnology, Department of Systems Biology, Technical University of Denmark, Denmark.*

Additional File 1

Table 1S. Amino acid sequence of PHO regulon gene products.

>Contig_New133_146: PhoR
VTTPVSLALAIGALVAGAVVGYLVARARTRREDVRPPGPTVAELYERLVRSSNNGVVVLN
RFGDMVLHNPRAYELGLVKVNQADPRARKAAEQVVETDEPMEIDLSPLEARGRQPEAVL
GQVRPLGDGFTVVEAVDHSEAIRLEAVRRDFVANVSHELKTPVGAIALLTEAVLDAAEDV
EEVRRFGGKILRESTRLGQLVTELIALSRLQGAERLPDLNVVEVDAVVREALGRTTLSAESA
DIRITTDTPSGLLIEGDRTLLVTALSNLLENAVAYSPAGSPVSISRRLNDGVVEIAVTDRGIGI
HEDEQTRVFERFYRADKARSRATGGTGLGLAIVKHVAANHGGSVGLWSRPGTGSTFTLRI
PAHIGPEHAAEPAKAGKQTSPAPRQEKIPERTQRLVVTGQDSPDHGGNL

>Contig_New133_147: PhoP
VTRVLIVEDEESFADPLAFLLRKEGFTAAVAGTGQAALEEFDRNGADIVLLDLMLPGMSGT
DVCKQLRQRSAVPVIMVTARDSEIDKVVGLELGADDYVTKPYSARELIARVRAVLRRGGE
PGSEGELAPLVLSAGPVRMDVERHVVTVDGAEVSLPLKEFDLLEYLLRNVGRVLTRGQLID
RVWGADYVGDTKTLDVHVKRLRSKIEPDPGSPRHLVTVRGLGYKFET

>Contig_New11_4: PhoD
MTEPTHSRRSLLKAGLTGASAVAAGLVLPSTAFAAAPLVRGGRPVLTHGVQSGDVTPGSAI
VWSRADRPSRLVVEIARDPSFRHARRVPGPLVGPDSGGTGSVRVAALQPGTEYHYRVTAE
ALDGRTTSEPLTGRFATAPVGRRDTRILWSGDVAGQNWGINPALGGMTIFSAMAARCPDL
FLHSGDTVYSDGPLTESVTLPGGRTWHNVVTPEKSKVAETLDEFRGQHAYNRLDGNFKRF
AAQVPVYAQWDDHEVLNNWYPGKILDNPAYTEKRVDVLAPRAYQAFHEWHPIDSRQAV
DGRVYRSFRYGSRAEIFVLDMRTYRNANTADQTKPGYILGDAQARWLADALGRSTATWK
IVQADMPLGLVVPDGTAIEAVANNLPGAPGGRETELAWVLREIQRRRVRNVVWLTADVH
YTAAHHYSPDRAAFQDFDPFWEFVSGPLNAGAFGPNQLDPTFGPEAVFVHAPPAANTSPID
GFQHFGELNIDGATGDLTVDLRDATGASLWSKKLHPQGR

>Contig_New140_38:PstS
MSGSGAAARRDDPVKLLMEETAVKIMRPLSAVGIVASAALVLAACGSDPAATNSSSSNSA
SAPAATGTADVECGGKSPLSAEGSSAQKNAIDIFTQQYSKKCSGQQVNYNPSGSGAGVKQ
FNANQIDFGGSDSPIKDADAAAAKTRCGSDAWNIPMVVGPIAIAYKLSGVDKLTLTPSVAA
KIFSGGITKWNDPAIKAVKGNESLNLPDKPIQVVSRADESGTTDNFQKYLGAAAKADWTK
GAGKKFNGGVGNGAQGSNGVATAVKASDGGITYVEGAFAKDGLTPALIDSGSGGVELNA
ANVAKSLDAAKFLKDGTNDLALDLNGIYASNTAGAYPLLLTTYEIVCSKYSNPDVAKAVK
AFLTVAATDGQQPLSAKGYVPIPQSLQTKVLAAVKAIS

>Contig_New68_128: Ppk
VSTDDGSTPAPRRRRNPANGSSETAKKTAARSSTAKPATAKPAAAKKVPSKSTARDTAAR
ATAGKARTRKTTSAATTPATTRRRSATHGNPRGAEEFRAVPSAPPAVTAAPTAVETLPDDR
YFNRELSWQDFNARVLALAEDESQPLLERTKFLAIFASNLDEFYMVRVAGLKRRDETGLL
VRSADGLTPREQLDYISKRNQDLVERQTGAFEKHLRPQLAEHDIRIVGWTDLSGADQLRLS
SYFSEQIFPVLTPLAVDPAHPFPYISGLSLNLAVTVRDPEGGTERFARVKVPSNVPRLMRVE
NERTSRTATFLPLEELISAHLGELFTGMDVTEHHVFRVTRNADFEVDEDRDEDLLQALERE
LAQRRFGPPVRLEVAQDMSEHMLELLLRELDVDPADVVEVPGLLDLTCLHQLSGVDRKEL
KDRPFVPATHPAFGERETPKSVFATLREGDVLVHHPYDSFSTSVQRFIEQAAADSKVLAIKQ
TLYRTSGDSPIVDALIDAAEAGKQVVALVEIKARFDEQANITWARTLERAGVHVVYGLVG
LKTHCKVSMIVRQEGSTIRRYCHIGTGNYNPKTARLYEDIGLFTADPSIGADVTDLFNVLTG
YSRQDTYRTILTSPHGIRRGIVRAIGEEIELARAGQQAGIRIKCNSLVDEQIIDALYHASQAG
VPVEIVVRGICTLKPGVEGLSENIHVRSILGRFLEHSRVFHFRAGGTHWIGSADMMHRNLD
RRIEALVRVKDPKLTAQLDNIFDSALDPETRCWVLTASGEWTPFPAGGSRVRDHQAELAK
LHGAAG

Table 2S. Best matching hits from BLAST analysis of Pho regulon gene products against SwissProt database.

>Contig_New133_146: PhoR

trD8HXM1
Two-component system histidine kinase [*senX*] [*Amycolatopsis mediterranei* (strain U-32)] 399 AA
Score = 677 bits (1748), Expect = 0.0
Identities = 355/387 (91%), Positives = 361/387 (93%), Gaps = 1/387 (0%)

trD9V0L7
Phosphate regulon sensor kinase PhoR[*Streptomyces sp.*AA4] 412 AA
Score = 627 bits (1618), Expect = e-178
Identities = 335/390 (85%), Positives = 348/390 (89%), Gaps = 8/390 (2%)

trD9VR28
Phosphate regulon sensor kinase PhoR [*Streptomyces sp.C*] 412 AA
Score = 261 bits (666), Expect = 2e-67
Identities = 169/366 (46%), Positives = 213/366 (58%), Gaps = 12/366 (3%)

trC0KWX8
PhoR (Fragment) [*Streptomyces clavuligerus* ATCC 27064] 458 AA
Score = 258 bits (660), Expect = 8e-67
Identities = 171/371 (46%), Positives = 216/371 (58%), Gaps = 20/371 (5%)

trQ0RS33
Sensor histidine kinase/phosphatase of two-component system for pho regulon (Partial match) (EC 2.7.3.-) [*phoR*] [*Frankia alni*] 464 AA
Score = 254 bits (649), Expect = 1e-65
Identities = 161/350 (46%), Positives = 204/350 (58%), Gaps = 29/350 (8%)

>Contig_New133_147: PhoP

trD8HXM2
Two-component system response regulator [*regX*] [*Amycolatopsis mediterranei* (strain U-32)] 229 AA
Score = 445 bits (1144), Expect = e-123
Identities = 227/229 (99%), Positives = 229/229 (100%)

trD9V0L8
Phosphate regulon transcriptional regulatory protein PhoB [SSMG_00202] [Streptomyces sp. AA4] 229 AA
Score = 438 bits (1126), Expect = e-121
Identities = 224/229 (97%), Positives = 226/229 (98%)

trB1VS84
Putative two-component system response regulator [*phoP*] [*Streptomyces griseus* subsp. griseus] 226 AA
Score = 359 bits (921), Expect = 2e-97
Identities = 178/228 (78%), Positives = 203/228 (89%), Gaps = 3/228 (1%)

trD9VR29
Phosphate regulon transcriptional regulatory protein PhoB [*Streptomyces sp. C*] 226 AA
Score = 359 bits (921), Expect = 2e-97
Identities = 179/228 (78%), Positives = 204/228 (89%), Gaps = 3/228 (1%)

trC7C2T9
PhoP protein [*phoP*] [*Streptomyces sp.* ATCC 55098] 223 AA
Score = 355 bits (910), Expect = 3e-96

Identities = 176/225 (78%), Positives = 200/225 (88%), Gaps = 3/225 (1%)

trQ1RPQ4
PhoP protein [*phoP*] [*Streptomyces natalensis*] 223 AA
Score = 354 bits (908), Expect = 5e-96
Identities = 176/225 (78%), Positives = 201/225 (89%), Gaps = 3/225 (1%)

trC5C8X1
PhoP-like protein [*Micrococcus luteus*] 226 AA
Score = 353 bits (907), Expect = 6e-96
Identities = 178/228 (78%), Positives = 202/228 (88%), Gaps = 3/228 (1%)

trQ83UZ0
Regulatory protein [phoP] [*Streptomyces lividans*] 223 AA
Score = 353 bits (906), Expect = 8e-96
Identities = 176/225 (78%), Positives = 201/225 (89%), Gaps = 3/225 (1%)

trQ82GC5
Putative two-component system response regulator [*phoP*][*Streptomyces avermitilis*] 223 AA
Score = 352 bits (904), Expect = 1e-95
Identities = 175/225 (77%), Positives = 201/225 (89%), Gaps = 3/225 (1%)

trC9ZH27
Phosphate two component system response regulator [*phoP*] [*Streptomyces scabies*] 223 AA
Score = 352 bits (904), Expect = 1e-95
Identities = 175/225 (77%), Positives = 201/225 (89%), Gaps = 3/225 (1%)

trC6R7T4
Alkaline phosphatase synthesis transcriptional regulatory protein *phoP* [*Corynebacterium tuberculostearicum* SK141] 235 AA
Score = 345 bits (885), Expect = 2e-93
Identities = 179/234 (76%), Positives = 195/234 (83%), Gaps = 6/234 (2%)

>Contig_New11_4: PhoD

trD8I1Q3
Phosphodiesterase/alkaline phosphatase D [*phoD*] [*Amycolatopsis mediterranei* (strain U-32)] 518 AA
Score = 1003 bits (2593), Expect = 0.0
Identities = 485/518 (93%), Positives = 494/518 (95%), Gaps = 1/518 (0%)

trD9V966
Phosphodiesterase/alkaline phosphatase D [*Streptomyces sp. AA4*] 542 AA
Score = 877 bits (2266), Expect = 0.0
Identities = 427/520 (82%), Positives = 463/520 (89%), Gaps = 4/520 (0%)

trA4F9J7
Phosphodiesterase/alkaline phosphatase D (EC 3.1.4.1) [SACE_1400] [*Saccharopolyspora erythraea* (strain NRRL 23338)] 519 AA
Score = 710 bits (1832), Expect = 0.0
Identities = 344/509 (67%), Positives = 396/509 (77%), Gaps = 1/509 (0%)

trQ0S552
Probable alkaline phosphatase [*Rhodococcus sp.*(strain RHA1)] 512 AA
Score = 603 bits (1554), Expect = e-170
Identities = 302/512 (58%), Positives = 361/512 (70%), Gaps = 4/512 (0%)

>Contig_New140_38:PstS

trD8HKR9
Periplasmic substrate-binding component of ABC-type phosphate transport system [*pstS*] [*Amycolatopsis mediterranei*(strain U-32)] 376 AA
Score = 620 bits (1600), Expect = e-176
Identities = 322/376 (85%), Positives = 329/376 (87%)

trD9V821
Phosphate ABC transporter, phosphate-binding protein PstS [*Streptomyces sp. AA4*] 373 AA
Score = 545 bits (1403), Expect = e-153
Identities = 281/373 (75%), Positives = 300/373 (80%)

trC6WPR6
Phosphate ABC transporter, periplasmic phosphate-binding protein precursor [Amir_6821] [*Actinosynnema mirum* (strain ATCC 29888 / DSM 43827 / NBRC 14064 / IMRU 3971)] 373 AA
Score = 369 bits (946), Expect = e-100
Identities = 202/378 (53%), Positives = 249/378 (65%), Gaps = 7/378 (1%)

>Contig_New68_128: Ppk

trD8HNI8
Polyphosphate kinase (EC 2.7.4.1) [*ppk*] [*Amycolatopsis mediterranei* (strain U-32)] 790 AA
Score = 1329 bits (3439), Expect = 0.0
Identities = 671/711 (94%), Positives = 679/711 (95%)

trD9VDU5
Polyphosphate kinase [*Streptomyces sp. AA4*] 808 AA
Score = 1273 bits (3293), Expect = 0.0
Identities = 639/705 (90%), Positives = 661/705 (93%)

trC7MXF9
Polyphosphate kinase (EC 2.7.4.1) (ATP-polyphosphate phosphotransferase) (Polyphosphoric acid kinase) [*ppk*][*Saccharomonospora viridis* (strain ATCC 15386 / DSM 43017 / JCM 3036 / NBRC 12207 / P101)] 743 AA
Score = 1120 bits (2897), Expect = 0.0
Identities = 554/683 (81%), Positives = 609/683 (89%), Gaps = 3/683 (0%)

trC6WG45
Polyphosphate kinase (EC 2.7.4.1) (ATP-polyphosphate phosphotransferase) (Polyphosphoric acid kinase) [*ppk*][*Actinosynnema mirum* (strain ATCC 29888 / DSM 43827 /NBRC 14064 / IMRU 3971)] 712 AA
Score = 1065 bits (2754), Expect = 0.0
Identities = 524/681 (76%), Positives = 593/681 (87%)

trA4FMN8
Polyphosphate kinase (EC 2.7.4.1) [*ppk*] [*Saccharopolyspora erythraea* (strain NRRL 23338)] 759 AA
Score = 1034 bits (2673), Expect = 0.0
Identities = 520/679 (76%), Positives = 572/679 (84%)

Table 3S. ***Amycolatopsis balhimycina*** **DSM5908 proteins differentially expressed under LP and LG conditions.**

Protein upregulated in LP

Spot	Protein	Gene acronym	Main metabolic pathway/Cellular function (category)[1]	Sequence source[2]	Theoretical pI/Mr	Experimental pI/Mw	Expression profile	Peptides[3]	Sequence coverage (%)	Mascot Score
113.1	3-isopropylmalate dehydratase large subunit	LeuC	Amino acid metabolism	Contig_New68_123	5.29/51	5.29/53	1.6 (P<0.05)	Protein identified by gel-matching [23-24]		
40	aminotransferase	Gabt1	Amino acid metabolism	Contig_New125_37	5.40/46	5.69/41	2.0 (P<0.05)	19	50	265
42	1-pyrroline-5-carboxylate dehydrogenase	PutA	Amino acid metabolism	Contig_New34_11	5.63/58	5.98/55	1.5 (P<0.05)	14	31	178
39	branched-chain amino acid aminotransferase#	IlvE	Amino acid metabolism	Contig_New89_185	5.51/50	5.84/38	1.8 (P<0.005)	14	37	189
29	Putative UTP-glucose-1-phosphate uridylyltransferase	GalU	Amino sugar metabolism	Contig_New129_4	5.04/33	5.15/30	1.7 (P<0.005)	8	42	126
127.1	phosphoglucosamine mutase	GlmM	Amino sugar metabolism	Contig842_8	4.97/45	5.11/45	1.7(p<0.05)	Protein identified by gel-matching [23-24]		
129.1	4-hydroxyphenyl pyruvate dioxygenase	HmaS	Balhimycin biosynthesis	Contig_New18_16	4.82/38	4.80/37	1.5 (P<0.05)	Protein identified by gel-matching [23-24]		
131.1	glycosyltransferase	BgtfB	Balhimycin biosynthesis	Contig_New18_8	5.10/42	5.27/44	1.8 (P<0.05)	Protein identified by gel-matching [23-24]		
35	4'-phosphopantetheinyl transferase	AcpT	Balhimycin biosynthesis	Contig_New129_94	5.67/25	6.17/29	2.8 (p<0.001)	8	53	103
133.1	4-hydroxy-3-methylbut-2-en-1-yl	GcpE	Biosynthesis of steroids	Contig_New63_69	5.51/41	5.14/40	1.7 (P<0.01)	Protein identified by gel-matching [23-24]		

	diphosphate synthase									
34.1	enoyl-ACP reductase	FabI	Fatty acid metabolism	Contig_New120_88	5.05/27	5.23/27	1.5 ($p<0.05$)	Protein identified by gel-matching [23-24]		
70	acyl-CoA synthase	L-ACS1	Fatty acid metabolism	Contig_New80_94	4.99/56	5.09/49	1.6 ($P<0.05$)	13	40	152
75	triosephosphate isomerase	TPI	Glycolysis/Gluconeogenesis	Contig_New116_115	5.69/28	6.09/28	1.9 ($p<0.05$)	11	53	217
28	phosphoglycerate kinase	Pgk	Glycolysis/Gluconeogenesis	Contig_New116_116	4.76/42	4.73/40	2.00 ($p<0.005$)	29	78	403
46	phosphoglycerate kinase	Pgk	Glycolysis/Gluconeogenesis	Contig_New116_116	4.76/42	4.68/40	2.00 ($p<0.005$)	22	73	336
47	phosphoglycerate kinase	Pgk	Glycolysis/Gluconeogenesis	Contig_New116_116	4.76/42	4.78/40	1.8 ($p<0.01$)	22	73	336
51	phosphoglycerate kinase	Pgk	Glycolysis/Gluconeogenesis	Contig_New116_116	4.76/42	4.45/26	2.2 ($p<0.005$)	9	39	127
64	glyceraldehyde 3-phosphate dehydrogenase	GAPD	Glycolysis/Gluconeogenesis	Contig_New116_117	9.44/51	5.39/33	1.5 ($p<0.05$)	18	52	266
65	glyceraldehyde 3-phosphate dehydrogenase	GAPD	Glycolysis/Gluconeogenesis	Contig_New116_117	9.44/51	5.14/33	1.8 ($p<0.005$)	18	52	266
66	glyceraldehyde 3-phosphate dehydrogenase	GAPD	Glycolysis/Gluconeogenesis	Contig_New116_117	9.44/51	5.27/33	1.5 ($p<0.001$)	15	48	210
24	glucose-6-phosphate isomerase	PgiA	Glycolysis/Gluconeogenesis	Contig_New51_10	4.80/56	4.75/57	1.8 ($p<0.005$)	28	64	428
139.1	fructose-bisphosphate aldolase	FDA	Glycolysis/Gluconeogenesis	Contig_New142_248	5.02/38	5.21/29	1.5 ($p<0.05$)	14	46	214
45.1	pyrophosphate-dependent-fructose 6-phosphate 1-phosphotransferase	P-PFK	Glycolysis/Gluconeogenesis	Contig_New151_106	5.42/36	5.27/33	1.5 ($p<0.05$)	Protein identified by gel-matching [23-24]		
46.1	Enolase	ENO	Glycolysis/Gluconeogenesis	Contig804_20	4.53/45	4.47/46	1.6 ($p<0.01$)	Protein identified by gel-matching [23-24]		
72	methylmalonate-semialdehyde	MSDH	Propanoate metabolism	Contig882_10	5.19/57	5.36/49	1.5 ($p<0.05$)	16	45	225

	dehydrogenase									
20	ferritin family protein	FFP	Iron metabolism	Contig_New94_6	4.86/21	5.00/21	>10 (p<0.0005)	8	45	516
165.1	inorganic pyrophosphatase	PPA	Oxidative phosphorylation	Contig_New42_75	4.98/19	5.06/23	1.5 (P<0.05)	5	39	192
15	NADH dehydrogenase I chain D	NuoD	Oxidative phosphorylation	Contig_New153_535	5,37/49	5.38/47	2.00 (p<0.001)	10	33	519
158.1	NADH dehydrogenase I chain E	NuoE	Oxidative phosphorylation	Contig_New153_534	4.73/31	4.65/36	1.5 (p<0.02)	Protein identified by gel-matching [23-24]		
174.1	thioredoxin	TrxR	Oxidoreduction	Contig_New142_31	4.56/12	4.62/10	1.5(p<0.03)	4	51	164
176.1	member of AhpC/TSA family	AhpC	Oxidoreduction	Contig_New63_2	4.58/17	4.49/16	1.6 (P<0.01)	Protein identified by gel-matching [23-24]		
176.1	member of AhpC/TSA family	AhpC	Oxidoreduction	Contig_New63_2	4.58/17	4.58/16	1.6 (P<0.01)	Protein identified by gel-matching [23-24]		
179.1	FMN reductase luciferase like	LUXL1	Oxidoreduction	Contig_New9_37	5.22/31	5.38/29	1.5 (p<0.02)	Protein identified by gel-matching [23-24]		
154.1	N5,N10-methylenetetrahydro methano-pterin reductase	LUXL2	Oxidoreduction	Contig_New68_87	5.44/29	5.34/28	1.7 (p<0.005)	Protein identified by gel-matching [23-24]		
181.1	transaldolase	TrA	Pentose phosphate pathway	Contig_New51_11	4.66/40	4.61/38	1.5 (P<0.05)	Protein identified by gel-matching [23-24]		
82.1	transketolase	TrK	Pentose phosphate pathway	Contig_New51_12	5.10/75	5.25/82	1.5 (p<0.05)	Protein identified by gel-matching [23-24]		
58.1	F420-dependent glucose-6-P dehydrogenase	G6PD	Pentose phosphate pathway	Contig_New87_62	5.35/37	5.63/37	1.5 (p<0.05)	Protein identified by gel-matching [23-24]		
198.1	elongation factor EF-Tu	EF-Tu	Protein biosynthesis, folding and modification	Contig_New153_434	5.12/44	6.27/23	1.6 (p<0.05)	8	18	108
59	elongation factor EF-Tu	EF-Tu	Protein biosynthesis, folding and modification	Contig_New153_434	5.12/44	5.36/25	1.8 (p<0.01)	9	23	125
78.1	peptidyl-prolyl cis-trans isomerase	PPI1	Protein biosynthesis, folding and modification	Contig_New94_114	5.67/18	6.27/15	1.8 (p<0.005)	8	65	127

289.1	peptidyl-prolyl cis-trans isomerase	PPI2	Protein biosynthesis, folding and modification	Contig_New94_90	5.04/13	5.18/17	1.8 (p<0.02)	Protein identified by gel-matching [23-24]		
195.1	Clp protease ATP-binding subunit	ClpB	Protein biosynthesis, folding and modification	Contig_New142_212	5.15/94	5.28/90	1.5 (p<0.005)	Protein identified by gel-matching [23-24]		
22	cold shock-like protein CspG	CSP-G	Protein biosynthesis, folding and modification	Contig_New133_66	8.98/9	5.59/9	>10 (p<0.0005)	4	34	205
62	chaperone protein dnaK	DnaK	Protein biosynthesis, folding and modification	Contig_New142_202	4.79/66	5.21/28	1.8 (p<0.01)	8	23	114
66.1	chaperone protein dnaK	DnaK	Protein biosynthesis, folding and modification	Contig_New142_202	4.79/66	4.76/63	1.5 (p<0.05)	Protein identified by gel-matching [23-24]		
77.1	trigger factor	TF	Protein biosynthesis, folding and modification	Contig_New89_24	4.38/51	4.31/63	1.5 (p<0.05)	Protein identified by gel-matching [23-24]		
71	GMP synthase	GuaA	Purine metabolism	Contig_New63_221	5.36/67	5.10/50	2.0 (p<0.005)	23	52	312
45	acetyl-coenzyme A synthetase	ACS	Pyruvate metabolism	Contig_New42_138	5.18/72	5.21/69	2.2 (p<0.005)	14	25	205
185.1	acetyl-coenzyme A synthetase	ACS	Pyruvate metabolism	Contig_New42_138	5.18/72	5.26/69	2.0 (p<0.002)	21	36	284
43	succinate dehydrogenase flavoprotein subunit	SdhA	TCA cycle	Contig_New135_138	5.56/65	5.79/61	2.3 (p<0.005)	19	46	185
177.1	dihydrolipoamide dehydrogenase	LpdA	TCA cycle	Contig_New63_21	5.15/48	5.26/48	2.2 (p<0.005)	Protein identified by gel-matching [23-24]		
14	dihydrolipoamide acyltransferase	SucB	TCA cycle	Contig_New89_176	4.60/61	4.52/87	1.9 (p<0.05)	16	46	199
19	dihydrolipoamide dehydrogenase	LpdA2	TCA cycle	Contig_New89_177	5.91/51	6.02/50	1.5 (p<0.02)	22	54	301
225.1	malate dehydrogenase	MDH	TCA cycle	Contig446_4	4.97/34	5.15/30	1.7 (p<0.02)	14	54	242
222.1	aconitate hydratase 1	ACO	TCA cycle	Contig_New120_105	5.13/79	4.82/97	2.0 (p<0.002)	Protein identified by gel-matching [23-24]		
90.1	succinyl-CoA ligase beta chain	B-SCS	TCA cycle	Contig_New63_203	4.59/41	4.50/39	1.8 (p<0.005)	Protein identified by gel-matching [23-24]		
88.1	succinyl-CoA ligase alpha subunit	A-SCS	TCA cycle	Contig_New63_202	5.35/30	5.67/29	1.6 (p<0.01)	Protein identified by gel-matching [23-24]		

290.1	thiamine biosynthesis protein ThiS	ThiS	Thiamine biosynthesis	Contig_New153_127	5.28/10	5.53/12	3.2 (P<0.0005)	Protein identified by gel-matching [23-24]		
116.1	thiamine biosynthesis protein ThiC	ThiC	Thiamine biosynthesis	Contig_New138_132	5.51/61	5.74/59	2.2 (P<0.0005)	Protein identified by gel-matching [23-24]		
92.1	phage shock protein A	PspA	Transcriptional regulation	Contig_New14_52	6.15/33	6.83/32	1.9 (p<0.05)	Protein identified by gel-matching [23-24]		
49	transcription antitermination protein nusG	NusG	Transcriptional regulation	Contig_New153_467	4.21/29	4.15/37	2.2 (p<0.02)	10	44	185
60	metallo-beta-lactamase superfamily protein	MLSP	Unknown	Contig_New11_45	5.16/31	5.20/26	1.9 (p<0.01)	9	46	163
53	FHA domain containing protein	FDCP	Unknown	Contig_New120_141	4.80/16	4.51/23	2.8 (p<0.005)	8	83	153
21	heat shock protein	Hsp18_1	Unknown	Contig_New122_10	5.08/16	4.26/14	5.9 (p<0.005)	10	81	126
149.1	possible ATPase	ATPase	Unknown	Contig_New129_237	6.57/39	5.26/40	1.5 (p<0.05)	Protein identified by gel-matching [23-24]		
50	phenazine biosynthesis PhzC/PhzF protein		Unknown	Contig_New138_198	6,67/35	4.53/23	1.9 (p<0.05)	6	22	338
9	hypothetical protein	HP18	Unknown	Contig_New14_31	4.79/31	4.76/33	3.0 (p<0.005)	9	53	119
10	hypothetical protein	HP18	Unknown	Contig_New14_31	4.79/31	4.84/33	2.3 (p<0.01)	10	61	178
17	hypothetical protein Rv0966c/MT0994	HP19	Unknown	Contig_New142_66	9.82/37	6.44/28	2.2 (p<0.05)	9	30	113
30	putative peptidyl-prolyl cis-trans isomerase	HP20	Unknown	Contig_New153_197	5.25/11	5.52/10	8.3 (p<0.005)	7	76	152
6	cytochrome P450		Unknown	Contig_New81_23	5.23/45	5.32/41	2.9 (P<0.001)	23	52	248
7	cytochrome P450		Unknown	Contig_New81_23	5.23/45	5.41/41	2.1 (P<0.001)	23	52	248

Protein downregulated in LP

5.1	ABC transport system ATPase component		ABC transporter	Contig_New68_10	5.81/27	6.40/28	0.67 (p<0.01)	Protein identified by gel-matching [23-24]		

36	extracellular sugar-binding protein		ABC transporter	Contig_New137_39	5,69/48	6.24/32	0.67 (p<0.005)	13	31	624
16	inosine-5'-monophosphate dehydrogenase	GuaB2	Purine metabolism	Contig_New63_232	5.59/52	5.87/56	0.36 (p<0.005)	10	26	621
124.1	adenosylhomocysteinase	SahH2	Amino acid metabolism	Contig747_8	5.28/55	5.52/50	0.59 (p<0.02)	Protein identified by gel-matching [23-24]		
27	alanine dehydrogenase	NAD	Amino acid metabolism	Contig836_18	6.01/43	5.95/29	0.62 (p<0.05)	8	27	116
25	acyl-CoA synthetase	L-ACS2	Fatty Acid metabolism	Contig_New129_97	5.31/59	5.61/55	0.46 (p<0.005)	10	19	120
5	glycerol kinase	GlpK	Glycerolipid Metabolism	Contig_New135_68	4.85/55	4.85/45	0.67 (p<0.02)	27	59	306
68	60 kDa chaperonin	GroEL	Protein biosynthesis, folding and modification	Contig_New138_148	4.85/57	5.01/47	0.30 (p<0.002)	12	30	145
48	30S ribosomal protein S1	RpsA	Protein biosynthesis, folding and modification	Contig_New79_28	4.77/58	4.55/57	0.48 (p<0.01)	19	35	292
41	inosine-5'-monophosphate dehydrogenase	GuaB2	Purine metabolism	Contig_New63_232	5.59/52	5.91/56	0.71 (p<0.05)	17	69	237
69	hypothetical protein	HP21	Unknown	Contig_New110_24	5.18/62	4.92/57	0.22 (p<0.0001)	10	29	145
8	hypothetical protein	HP16	Unknown	Contig_New94_58	6.07/26	5.94/32	0.45 (p<0.005)	11	72	167

[1]Functional classification was realized according to BioCyc, KEGG and Expasy metabolic pathway databases. [2]Referring to data from genome sequence (Tab. 3S). [3]Number of peptides identified by MALDI-TOF PMF or nLC-ESI-LIT-MS/MS analysis. Gel-matching identification was performed automatically using *A. balhimycina* protein 2D reference-maps (http://www.unipa.it/ampuglia/Abal-proteome-maps) [23-24] and Image Master 2D Platinum software 7.0 as described in Materials and Methods.

Tab. 4S. Amino acid sequence of the MS-identified *Amycolatopsis balhimycina* DSM5908 proteins.

>Contig_New11_45
VRDVPENHREDGDDAEEVPADPARRRRGGHGRPGVGWRGRPEAYVASRPGACAGGTAW
DETTTVCTVNIVETYTGHVDPGGDATRRTLEALTITKLSVGPMDNNTYLLVCRESNEALLV
DAANDPDRISDLIGHGPDRPSLKTVVTTHQHQDHWQALGAVAGANGANTAAHPLDASPL
PVPPDFLVEQGGTLSVGRVTLSVIHLRGHTPGSIALLYRDPAGIPHLFTGDSLFPGGVGKTAS
PENFTSLLDDVESRIFAELPDETWFYPGHGDDSTLGAQRPNLKEWRERGW

>Contig_New14_21
MSEEQTTPAEEPNDELVRVDEFETDVPLELDVSVTIGRVEIVLEGDSGARVELRHDQGEQQ
PWVAGVNNLLSWVGERFGDQLGVDPAASPAEAVRQSRIEKLGNRLVVSAPKAWQLRNVA
LAVKVHAPAGSHVEVRAGAADVTVTGSAGRVDLLTGSGEVKLDRADGSATIRTGSGGVK
LGPTLGGLQLRSGSGHVEASSIAGSATLATGTGDVWLGAVSGEVMARTGSGDLSVADAAS
GSLDLITGSGEVRIGIRGGTAAEVDLTSSAGRVSSELDVADAAPEGGVKLKVRARTGTGNA
VVTRAAG

>Conitg_New14_31
MSEEQTTPAEEPNDELVRVDEFETDVPLELDVSVTIGRVEIVLEGDSGARVELRHDQGEQQ
PWVAGVNNLLSWVGERFGDQLGVDPAASPAEAVRQSRIEKLGNRLVVSAPKAWQLRNVA
LAVKVHAPAGSHVEVRAGAADVTVTGSAGRVDLLTGSGEVKLDRADGSATIRTGSGGVK
LGPTLGGLQLRSGSGHVEASSIAGSATLATGTGDVWLGAVSGEVMARTGSGDLSVADAAS
GSLDLITGSGEVRIGIRGGTAAEVDLTSSAGRVSSELDVADAAPEGGVKLKVRARTGTGNA
VVTRAAG

>Contig_New34_11
VDAVTQTPAPTNEPVLTYAPGSAERAELEGALKRLGQAEPVDLTVTIGGEQRPGGGEQIDV
VQPHNHAHVLGTIHSATRQDATDAIAAAAKAAPEWRALSYDDRAAILLRAADLLTGKWR
ATLNAATMLGQSKTATQAEIDSACELADFWRFNVEFGRRILAEQPISSPGVWNRMEHRPLE
GFVYAITPFNFTAIAGNLPTAPALMGNTVLWKPSPTQSFAAHLTMRLLEEAGLPPGVINLLP
GDGKAVSEVALTHRDLAGIHFTGSTATFQHLWGTVGANIAGYRGYPRLVGETGGKDFVL
AHPSADVDVLRTALVRGAFEYQGQKCSAASRAYVPRSLWAKLKDGLVSETEALSYGDVT
DLSHFGGAVIDRRAFTKHSDLFDSVRDDASVEVLTGGTADDSVGYFVKPTILVSDNPKHEI
FSTEYFGPILSVHVYEDGDFDAVLKLVDETAAYALTGAIIANDRTAVAKASEALRFAAGNF
YVNDKPTGAVVGQQPFGGARASGTNDKAGSIFNLQRWTSPRSVKETFVPPTTVRYPHQG

>Contig_New42_138
MTEQSPALDNLLTESRTFPPSDEFAGQANAKADLYAEADADREAFWAKQAERLTWDTKW
TTVLDWTNAPFAKWFVGGKLNVAYNCVDRHVESGHGDQVAIHWVGEPGDTRDITYAEL
KTEVSRAANALVSLGVTAGDVVAIQLQMVPEAIFAMLACARIGVLHNVVFGGFSPTALRA
RVDDAAAKVVITSDGQFRRGKAAPMKANVDEALEGAETVEKVIVVKRTGDKLEGDVPWT
DGRDLWWHELVDGQSEEHTPEAFDSEHPLFILYTSGTTGKPKGILHTSGGYLTQTAYTHHN
VFDHKAGEDVYWCTADIGWITGHSYIVYGPLANRVTQVVYEGTPNTPHEGRHWEIVQKY
KVSLYYTAPTLIRTFMKWGAEIPEKYDLSSLRVLGSVGEPINPEAWIWYRENIGAGKTPIVD
TWWQTETGAIMISPLPGVTSTKPGSAQKALPGISAKVVDDQGNEVGPGGGGYLVLDKPWP
SMLRGVWGDEERFRDTYWSRFKDQGFYFAGDGAKYDNDGDVWLLGRVDDVMNVSGHR
ISTTEVESALVSHPTVAEAAVVGATDPTTGQGIVAFVILRGNAVDGGEEAIQALRNHVAKEI
GPIAKPRQIMVVPELPKTRSGKIMRRLLRDVAENRQVGDVTTLADSSVMDLISSGLKSGKS
EE

>Contig_New51_10
MTTGEKTGVEIVDVALAERAAPLAEELVAGQAASKLAAQDATLWGPDAEAEASIRLSWTS
LHKSSRPLIGEIEALRTELRSEGVDRVVLAGMGGSSLAPEVITATDGVALTVLDTTDPGQVA
DALAGDLDRTVIVVSSKSGGTVETDSHRRIFAKAFADAGIDAARRIVVVTDPGSPFQELSEK
EGYRKVFLADPHVGGRYSALTAFGLVPAGLAGADVARLLDQAASVAEELAADSADNPAV
KLAAAWAAAHETGAEKVVLADTGSGIKGFPDWAEQLIAESTGKQGTGLLPVAVEGAEAA
GFADAKSDATPTAVGSPQGAAKIAVTGSLGAQFLLWEFATALAGRLLGINPFDQPDVETA
KKAARALLDDPGKLKGGEEPSNVDGPVEIFGSEGVSTEGSLTDVLRAFFASAPDAGYIAVQ
AYLDRLDDASTVVLRGEIAKRTGKQTTFGWGPRFLHSTGQYHKGGHQNGVFLQLTGAVE
RDLDVPDRPYTLGQLQHAQALGDGQVLAEHGRPVLRLHLTDRAAGLAAVVRAVQEVTA

>Contig_New63_221
MVNGPVPGSSSDFRGRTVQQPATGSHRYSSSVSPSSFGRVCTFQWKSSWSVSSFVQFTAIPR
TVGRIEDSTGPESRVTRPTWDDGCRNPLLPGGRQVPSPTGPVLVVDFGAQYAQLIARRVRE
AQIYSEVVPHSASTEEILAKNPAAIILSGGPSSVYAEGAPGMDPKLTEAGVPMFGICYGHQL
LASALGGVVEPTGVREFGRTEVRVTGDGGVLHAGLPAHQPAWMSHNDSVTKAPEGSVVT
ASSDGAVVAGFEDVERRFAGVQYHPEVAHSPHGQEVLRRFLRDIAGIEPQWTTSSIVEEQIK
RISDQIGDGRAICGLSGGVDSAVAAALVQRAIGDRLTCVFVDHGLLRAGERTQVEQDFVSA
TGVNLITIDARERFLDALAGVTDPEQKRKIIGREFIRVFEQAERDLKAQGDYKFLVQGTLYP
DVVESGGGEGTANIKSHHNVGGLPDDLQFELVEPLRLLFKDEVRRVGLELGLPETIVQRQP
FPGPGLGIRIIGAVDQERLDTLRAADLIAREELTAAGLDRSIWQCPVVLLADVRSVGVQGD
GRTYGHPVVLRPVSSEDAMTADWTRLPYDVLERISTRITNEVPEVNRVVLDVTSKPPGTIE
WE

>Contig_New63_232
MTSDGTTAPVPAKFAMLGLTFDDVLLLPAESDVVPSAVDTSSRLTRNITLGVPLVSAAMDT
VTEARMAIAMARQGGIGVLQRNLPIDEQAAAVEVVKRSEAGMVTDPVTCAPDATLAEVD
ALCAKFRISGVPVTDAAGTLVGIITNRDMRFEVDHSRPVSEVMTKPPLITAQVGVSADAAL
GLLRRHKIEKLPIVDGAGKLRGLITVKDFVKTEQYPKATKDPDGRLIVGAAVGVGPDGHQ
RAMALADAGVDVLMVDTAHGHSRAVVDTVSLLKKELGDSVDIVGGNVATRAGAQALVD
AGADGIKVGVGPGSICTTRIVAGVGVPQISAIYEADLAARPAGIPVIGDGGIQYSGDIAKAIA
AGASTVMLGSLLAGTAESPGDLILVNGKQFKVYRGMGSLGAMQSRGQGKSYSKDRYAQD
DVLNEDKLVPEGIEGRIPFRGPLANVVHQLVGGLRAGMGYAGAETIAQMQEAQLVRITAA
GLKESHPHDITMTVEAPNYTTR

>Contig_New68_10
MSEPILEIKGLNKSFGPVHVLHDVDFDVRAGEVTALVGDNGAGKSTLVKCIAGIHPYDSGT
VRFNGQDAHIRGPRDAADLGIEVVYQDLALADNLDIVQNMFLGRERGNSWKLDEASMEK
AARETLASLSVRTVKSVRTPVSSLSGGQRQTVAIAKSVLWNSKVVVLDEPTAALGVAQTR
QVLDLVRRLAEQGLGVVLISHNMADVFEVADRIAVLYLGRLVTEVHTKDVTHGQVVELIT
AGRSGDLGLARPEAVVL

>Contig_New79_28
MLPSRRHIALTTANPRFPSTGATRLMTTDTAIAPTAPNAAPQVAINDIGSEEDFLAAIDKTIK
YFNDGDIVEGTIVKVDRDEVLLDIGYKTEGVIPSRELSIKHDVDPAEVVTVGDEVEALVLQ
KEDKEGRLILSKKRAQYERAWGTIEELKEKDEPVKGTVIEVVKGGLILDIGLRGFLPASLVE
MRRVRDLQPYVGRELEAKIIELDKNRNNVVLSRRAYLEQTQSEVRSEFLNALAKGQVRKG
VVSSIVNFGAFVDLGGVDGLVHVSELSWKHIDHPSEVVEVGQEVTVEVLDVDMDRERVSL
SLKATQEDPWRQFARTHAIGQIVPGKVTKLVPFGAFVRVEEGIEGLVHISELAERHVEIPEQ
VVQVNGDVMVKVIDIDLERRRISLSLKQANEGVTPDTEFDPTQYGMAAEYDAEGNYIYPE

GFDPDTQEWQEGFDKQREEWERQYAEAHTRYEAHMKQVVKAVEADAEAAADAATGIEG
GAESYTSSGSAPADTKSSGGTLASDEQLAALREKLSGGA

>Contig_New80_94
MPEDVRPRVRLFGELVAHWARERPADTALIFGDRSWTWAEFDERIRRLSGALTAAGITRG
DRVAFVDKNHPACLETTFAAAGIGAANAVVNWRLSGEELAYVLKDAGAKIVFVGAELVP
ALDAIRDRLPAVERVVVVGGDADEYESFLTSAEPHAGTGVDTDDGVLVMYTSGTTGFPKG
AVLTHRSVLAHGLAAGTAFPIGPGDVNLVAMPLFHVGGSCYAVSGFLYGEPSYLTREPDA
ASLFAALQAGITHAFLVPAVVAGIAQAGEAALTAFSRLRYLCYGASPMPLPLLRTVLAAWP
GVRFAQVYGMTELSGAVTALDPEAHRDDTRPERLASAGTALSGVDIRIADPVTGEDTEVG
EVWVRTEQRMAGYLGKPEATAETIVDGWVRTGDVGRLDDGGFLFLEDRVKDMIITGGEN
VYSPEVERVVAEFPGVAEVAVIGIPDDRWGEQVKAVVAGDQLDAEKIVEFCRERLAHYKC
PRSVDVVEALPRNATGKILKRSLREPYWRDRDRNV

>Contig_New81_23
MTTTPADTWGLHASQFWLRGKQPESRVEFAAELGMWNVYGHPEIEEILRDPATFSSDTTR
LVPKEMMPDADLEAMTAGNLLQLDPPLHNKMRKLVSRAFTPKVVADLEPRIAAITHEMLD
AVTTPGRLELVEDLAYPLPVIVIAELLGVPASDRHLFKGWVDKMFESSEQLSLVKKDEKQD
EAIKKSLEGQKSLTDYLGVHVDERRKQPREDLLTKLVEAELDGERLSRNEVVNFANILLLA
GHITTTMLLGNSVLCLDTHREWDERIRADRSLVPPAIEESLRFLTPFAAVARTTMREVELGG
VTVPADQLLMTWVAAANRDERVFTDPDAFDPLRDPNPHLAFGRGIHFCIGAPLARLEGRV
ALNILFDRYPALRTIPGEPPKFQVNPNMTGVRELPLTTA

>Contig_New89_176
MAYSVTLPELGESVTEGTVTRWLKQEGDTVEVDEPLLEISTDKVDTEVPSPVAGTVVKISA
QEDETVEVGGELAVIDDGSGGVPESESDTAPAQEEQKSEPEPEPEPEPQAQAAEESAPSKPD
TAPAAGGEGTEVKLPELGESVTEGTVTRWLKQVGDSVEVDEPLLEISTDKVDTEVPSPVAG
TVLEIRAGEDETVEVGGVLAVIGDANAAPKAESKPEPEPEPKPEPKPEPVQEAKPKPEPKPE
PQAAPAAKAPEAAPAAEAKDGSADGPYVTPLVRKLASEHGIDLASLTGSGVGGRIRKQDV
LAAAEEKQKAAAAPAPAAAAPAAAAAPSAPAARPAPAVSPELAALRGTVQKASRIRQITA
TKTRESLQIAAQLTQVQEVDVTKIAKLRQRAKAGFKEREGVNLTFLPFFAKATVEALKQHP
NVNASYNEDTKEITYHGAVHLGIAVDTERGLLSVVIHDAGELSLAGLAHRIADLAGRARA
GQIKPDELSGGTFSITNIGSVGALFDTPIIVQPQSGILGTGAVVKRPVVVADADGNDTIAVRS
MAYLPLTYDHRLVDGADAGRFLTTIKQRLEEGNFESELGL

>Contig_New89_177
VSRARLYPARAAPEHHPTACRGVNEVTDTSADLVILGGGSGGYAAAFRAAELGLSVTLIEK
DKLGGTCLHRGCIPTKALLHAAEVADETREAEAVGVKAVFEGIDIAGVNKYKDGIVARLY
KGLQGLAKAHKVNLVEGSGTFVGGTTVEVDGTRHTGKNVILATGSYSRTLPGLELGGRIIA
SEQALALDYVPKKVVVLGGGVIGVEFASVWASFGVDVTIVEALPRLVPNEDEFASKQLER
AFRRRKIAFKTGVRFTGAKQDDNGVSVSLESGETIEADLLLVAVGRGPNSAGHGYEEAGV
KIERGFVLTDERLRTNLPNVYAVGDIVPGLQLAHRGFQQGIFVAEEIAGQNPRVIDESGIPR
VTYSHPEVASVGLTESQAKDKYGSDVTTFTYDLGGNGKSQILKTSGGVKLVKAPDGPVVG
VHMVGDRVGELIGEAQLIYSWEAFPEDVAPLIHAHPTQTEALGEAFLALAGKPLHVHS

>Contig_New89_185
VDLPPPHDAEDAPDQAEEAAEAAVQHGPADHAEQADAEGQQHRGDERLDVGAGGLGPG
DRLSGVVGHSAILERSRRRCGMPGTQGRPTNAPPTKRYRLAMTTTTQFAHVPHPSPASADR
VAEVLAAPGFGVYFTDHMVTVKWSKAQGWHDAQVGPYAPFTLDPATSVLHYGQAIFEGL
KAYRQPDGSIASFRPDANATRFRQSAERLAMPQLPEDVFIESLRELVAVDSRWVPTRQGDS
LYLRPFMISTSTGLGVNSPAADYVYTVIASPAGSYFSGGVKPVSVWLSTEYVRAAPGGTGA

AKCAGNYAASFVAQAQAVEKGCDQVVWLDAVERRWVEEMGGMNLFFVFGSGENARLV
TPELTGSLLPGVTRKSLLQLASRLGYKVEERRISTDEWEKAAASGELTETFACGTAAVITPV
GHVKHANGEFTIADGQPGALTMKLREELTGIQDGTRPDADHWMVKLG

>Contig_New94_58
MSQHVFKRAGVLAATIGVAGLLGAGVASAHVTANVYGPQPTKGGYAAIVFRVPSEEPNT
MTTKVAVDFKADYGIGSVRTKPLPGWTAEVTKSKLPAPITKDNGTKITEAVTAVTWTAQP
GNELKATDYQEFSVSFGPLPTNVDEVEFPAHQTYSDGKVVDWNQPTPASGQEPEHPAPTV
KLAAKVEGDGDGDHAAMAATTGEHTEAAAATSDTTARWLGGAGLLVGAIGLGVGAGAT
IRARKATAKSGGNS

>Contig_New102_120
MRLGLAGTGRIGTAHAETLKGFEEVESVVVADVDTARAAAAAAKLGVESMSLDELFAAG
LDGLVVTAATDAHPGLIIAAVDAGIPVFCEKPVAADIPGTLAVIDRIDASDVPVQIGFQRRF
DAGYAAARAAVASGELGWLHTLRATTFDPAPPPAEYVAHSGGLFRDCGVHDFDIVRWVS
GREVTEVYAVGANRGERFFVDAGDVDTAAATLTLDDGTLATVSLTRYNGAGYDVRLDVL
GSVSGVVVGLDDRAPLRSVEPGVAPLPGPAYPGFMERFRPAYTTELRAFLDVVAGRAPSPC
VAANALEAFYIAEACEVSRRERRPVQLAEVRR

>Contig_New110_24
VRRMASTASEGVHGICDRYVDDYAAADPVAATAQGIAGHDHRLTDYSAAGFAERAGLAA
RAHAAVTAAEPRDAAERAAKAVFTERVGLELEIHEAGLDVASLNVIASPVQELRMVFDLM
PLETEHDWSVVATRLGEVPKALAGVRSGLLSAADAGRVAALRQVAKVAEQCETWAGLK
EETGFFTGLVGGASSQGEALKSDLAHGARAADEAYAEFAGFLRAELAPKAPVKDAVGED
VYRLWSRYFVGATLDLREAYEWGWAEFARIEEEMRAVANRVKAGATPAEAAAVLDADP
RYRVRGRAEFEAWMQRLSDQALESLRGKHFDISDRVMALECKIAPPGGGVGAYYTGPSED
FSRPGRMWWSLPAGRDEFVTWRETSTVYHEGAPGHHLQIATAVDQSDGLNKYQRMMAF
TSGHAEGWALYAERLMQELGYLADDGDLLGMLSEQLFRAARVIVDLGMHLELAIPSGTGF
HEGERWTPELGLEFVLSRTITDPAHVHDEIDRYLGWPGQAPAYKIGERLWLAARAEAQVR
AGKSFDIKHFHTEALKLGGMGLDTLREQLSQLD

>Contig_New116_115
VARKPFIAGNWKMNQNHLEAIALVQKIAFALPEKYYAKVDVAVLPPFTDIRSVQTLVDGD
KLSLTYGAQDIAPQDSGAYTGDISGLMLAKLGCKFVAVGHSERREYHAETDELVNKKVKA
ALKHGITPILCIGEKLEVREAGEHIHHTTTQLIDGLKGLKAEQVKDVVVAYEPVWAIGTGK
VASSSDAEEVCKAIRATLQEKYGDEVASSVRVLYGGSVKSGNISELVGCENIDGALVGGAS
LDGEEFTKLCALAAGGPLP

>Contig_New116_116
MSVKNLDDLLSEDGVGVQGRYVLVRSDLNVPLDGDRITDDGRVRAALPTIKKLADAGAK
VVVTAHLGRPKGEPDPKYTLAPVAKRLSELLGAEVALAGDLVGESAKALTGGLADGGVV
LLENVRFDARETSKDAVDRSELAAELGALVPGGAFVSDGFGVVHRKQASVYEVASVLPAY
AGGLVLAELDVLKKLTDDLQGPYVVVLGGAKVSDKLGVIANLLTKVDRLLIGGGMAYTF
LKAQGHEVGNSLLQADQLDQVKGFLAEAEKRGVELVLPVDVLAATGFAADAEHEVVAAT
AIPADREGLDIGPATRELFAGKLADAKTVFWNGPMGVFEFEAFSGGTRAVAEALVKSDAF
TVVGGGDSAAAVRQLGLPEDGFSHISTGGGASLEYLEGKELPGVTALEEKN

>Contig_New116_117
MRFRPEEAAGRPRRAAPGVAGSVESSTSRPAVEPPSAAPSQPAGARCGLNRSRCSADRRRP
ARHTRRSQPEPVPAPESRHPSGPPREPAVAVRFLNRFLTRSPSGGSLVRAVRDAHARTLGW
PRARKSRPGLRPILRGVAAVTVRVGVNGFGRIGRNFFRAVQASGHDIEVVAFNDLGDVAT

MAHLLKYDSILGRFPGEVSVSDEGIVVDGKTIKALAERDPANLPWGDLGVDVVVESTGFFT
NADAAKAHIAGGAKKVIISAPAKGEDLTIVLGVNDDKYDGSQNIISNASCTTNCLGPLAKV
LQDAFGIEQGLMTTIHAYTQDQNLQDAPHKDMRRARAAALNIVPTSTGAAKAIGLVLPEL
QGKLDGYALRVPVPTGSATDLTVTLTKAATLEEVNAAYKAAAEGPLSGYLRYNEDPIVSS
DIVTDPASCIYDAPLTKVIGNQVKVVGWYDNEWGYSNRLADLVKLVGSKLS

>Contig_New120_88
VPGLLEGKRLLITGIITDASLAFHAAKIAQQEGAKVVLTGFGRMSLVERIAKRLPEEAPVIEL
DVTNQEQLDGLADKVREHVDGLDGVLHSIGFAPQTCLGAPFLDAPAEDVKTAIEISTYSYM
SLAKACLPLLGRGASYVGMDFDARVAWPVYNWMGVAKAGLESVNRYLAKELGPRGIRV
NLVSAGPMKTMAAKSIPGFVDLEDGWGERAPLGWDSTDPDPVAKSVCAVLSDWLPATTG
SMIMVDGGVHFLGI

>Contig_New120_141
VSTNDGPGGVPPEQSPERTSVFRADFLAEAEGHESVPAPEAPVQGVDALPAGSALLVVKRG
PNAGSRFLLDRDTTSAGRHPDSDIFLDDVTVSRRHAEFRREGGEFVVIDVGSLNGTYVNRE
PVDQAVLAGGDEVQIGKFRLVFLTGPGHGGQGAQ

>Contig_New122_10
MLMRTDPFRELDRLTQQVFGTPGTWSKPTAMPMDAYRAGDEFVVCFDLPGVSPDAIELDI
ERNVLTVKAERRPLPGGDDVQMQVSERPLGVFSRQLFLGETLDTDHIAAGYEAGVLTLRIP
VAEKAKPRRIEIGGAQSDRKEIKA

>Contig_New125_37
MTDELLARHRAVMPSWMSLLYEEPIEIVHAHDRRMTDAQGRTYLDFFAGVLTNSMGYDV
AEIGDAVRKQLDTGILHTSTLYLIRSQVELAERIAKLSGIPDAKVFFTNSGSEANDTALMLA
TQFRRSNQVLAMRNSYHGRSFGTVAITGNRGWSASALSPVKVSYVHGGYRYRSPFRDMS
DADYIDACVADLVDVLATATAGDVACLIAEPIQGVGGFSLPPDGLFRAMKEVLDEYGVLFI
SDEVQTGWGRTGEHFWGIEAHGVTPDMMTFAKGLGNGLAVGGVVARGDVLDCFQAQSF
STFGGNPVSMAGATAVLDYIKDHDLQANCAARGAQLLSGLRAFDCPLVAEVRGKGLMIG
VELIKPGTTTPNVAAAARMLEETKKRGLLIGKGGLHGNVLRLGPPMTLTADEAQEGLDILV
DSLTATHAALS

Contig_New129_4
MHDLTLGFAPMTGAATTQTFRTAIVPAAGLGTRFLPATKAVPKELLPVVDTPGIELVAAEA
AAAGAERLVIVTSPGKDAVVKYFEKQPELEQNLEAKGKTELLAKVRRGSELLAVETAIQE
QALGLGHAVAQAEPNLKPDDEAVAVLLPDDLVLPTGVLDRMSAVRAQYGGSVLCAFDIP
KAEISPYGVFDVTDTDDEDVKRVHGMVEKPKPEDAPSTYAAAGRYLLDRAIFDALKRITPG
SGGELQLTDAVALLISEGHPVHVVVHRGGRHDLGNPGGFLRAAVDFALETPEYGPSLRAW
LTERLGTERP

>Contig_New129_94
MEIVVRWSSPLPAEERFLRLLDDVEQGRFAAYRQEADKRRFLTGRVLAKTVAAERLGVPV
ESVKFDATCEDCGKPHGRPRIPGADLALSISHSGDLIGLAATPSIPVGLDVETTTRRADEGLI
EYALSPAETASLAGLPAEDRAAAFFVYWTRKEAVMKATGKGLRIPLKSITFSRHDEPARLV
FSGDAALNPATTRLADLKAADGYRAAIAVLTTDELSVTEEHWTP

>Contig_New129_97
MLSTMQDGQLSLANLLRHGTSVHSASEVITWTGSEARRETYGDLGRHAARLANALRSLGV
TGDQRVGTFMWNNAEHMAAYLAVPAMGAVLHTLNIRLFPEQLVFVANHAEDHVVIVDG
TLVPLLAKQLPQFKTVRHVIVANGDAASLEAPDGVEVHSYAELLAAQPDTFDWPDVDERS

AAAMCYTSGTTGDPKGVAYSHRSIWLHSMQVCMTDSMKLAQHDKALAIVPMFHAMAW
GLPYASLMVGASLLMPDRFLQPAPISAMLAAEKPTFAGAVPTVWQGLLAHLEAHPQDISH
LREVVVGGSAVPPSLMHAFQERHDVPILHAWGMTETSPLGSVARPPASATGDDIWKYRYT
QGRFPASVRARLIDDDGAVLPWDNEAVGELEVQGPWIAASYYGGAEVDPDKFHDGWLRT
GDVGKISPDGFLTLTDRAKDVIKSGGEWISSVDLENQVMGHPAVAEAAVVGIPDEKWDER
PLVAVVLKEGQSVTPEELRDYLSDKVAKWQLPENWTFVDEVPKTSVGKFDKKRIRASYSE
GKLDIAQL

>Contig_New129_237
MPAPRPPFSRPHARAGPRPPSWPRWPPPTSGPKPPNWTLSPWAVVEYLLGGTLADGTVITP
KYVGPRRLIEVAVATLATDRALLLLGVPGTAKTWVSEHLSAAISGDSTLLVQGTAGTSEESI
RYGWNYARLIAEGPSTAALVESPVLRAMRDGKLARLEELTRIPADVQDSLITILSEKTLPVP
ELGTEVQARPGFNLIATANNRDKGVNELSSALRRRFNTVVLPLPDSAEAEVEIVSRRVAEL
GASLKLPIEAAELAEIRRVVTVFRELRSGRTEDGRTAVKSPSGTLSTAEAISVLTGGLALAT
HFGDGVLRPHDVAAGIHGAVVKDPVADRAIWIEYLETVVRERDGWADFYRAGQELS

>Contig_New133_66
VPLRWHSKRICVTRSTAVAQGTVKWFNAEKGFGFIAQDGGEGDVFVHYSEIEGRGFRTLE
ENQRVEFEVGQGQKGPQAQKVRAI

>Contig_New135_68
MTSYVAAIDQGTTSTRCMIFNHEGRVVSVDQREHEQIFPKAGWVEHNAEEIWENTRRVAA
GALAKADLTAKDIAAVGITNQRETALVWDKTTGTPVYNAIVWQDTRTDRIVTELGNLGGG
QERYRAKVGLPLATYFSGPKVKWILDNVEGAREKAEAGDLIFGNMDTWVLWNMTGGVD
GGIHVTDPTNASRTMLMDLDTLQWDAEIAGDMGIPLSMLPEIRSSSEEYGKVREKGALAG
VPIAGILGDQQAATFGQACLSPGEAKNTYGTGNFMLLNTGTEKVMSENGLLTTVCYKIGS
NDTIYALEGSVAVTGSLVQWLRDNLGLITTAAEIEEHARSVEDNGGAYFVPAFSGLFAPYW
RSDARGAIVGLTRFVNKGHLARAVLEATAFQSREVIDAMNADSGVPLKSLKVDGGMVVN
ELLMQFQADILGVPVIRPVVNETTALGAAYAAGLAVGFWKSEDDIRTNWAQDKQWDPAM
DDSRREREYRNWKKAVTKTFDWVDDQD

>Contig_New135_138
MQFHKYDVVIVGAGGAGMRAAIESGQRARTAVLTKLYPTRSHTGAAQGGMCAALANVE
EDNWEWHTFDTIKGGDYLVDQDAAEIMAKEAIDAVLDLEKMGLPFNRTPEGKIDQRRFGG
HTRDHGKAAVRRACYAADRTGHMILQTLYQNCVKYGTEFFNEFYVLDLVTTPDENGNPV
ASGVVAYELATGELHVFQAKSIVMATGGAGKIFKTTSNAHTLTGDGLGIIFRKGLPLEDME
FFQFHPTGLAGLGILISEAVRGEGGILRNASGERFMERYAPTIKDLAPRDIVARSMVQEVLQ
GRGCGPNKDYVVLDVTHIPEETLNAKLPDIMEFSRTYLGVDPVKEPVPVFPTCHYVMGGIP
TNIHGEALRDNENVIPGLYAAGEVACVSVHGSNRLGTNSLLDINVFGRRAGIAAAEYALAH
EHVELPSDPTTLVEEQLAGLLSEHGDERVADIRKEMQQTMDSHASVYRTEDTLKQALTDIQ
ALKERYQRITVSDKGKRYNTDLLEAVELGFLLELAEVLIVGALARKESRGGHAREDYPTRD
DTNFMRHTMAYKQGAGLSSDIRLDYKPVTFTRYEPMERKY

>Contig_New137_25
MSVHPSRRRMCGAVLTAVAATTLVTGCGSSVGGSAADSTLVSYTGQSGDYQINFNPFSSST
MEGPGTIFEPLFFYNITQDAKPLPLLGTDFAWNADGTQLLVTLRPNVKFSDGTPFTAKDVA
FTLDMVAKNKTINTTGYDGKAVATDDTHVKITFSKPAFMQGAQVLGKTFIVPQHLWSKIP
DPANDVIAQPVGTGPFVLEEFKPQAFTFKANTGYWGGEPAVKRIRYLALAGNQSGADALK
SKQIDWQTGPVPDIKNVAKNYPGYQAITVPMNQMNLTTCSNAALGCTGPQTDPAVRKAIY
YAINRTQLNSLAFEDTASDISPGFTLLGRDAKYVSPKLQEKLAPKSPDLTRSAGLLQGAGY
AKGPDGLFAKDGKPLELTVQVPSGWTDYITAINTMAQQLLPAGIKLLAQQVSYNEWADAR

VRGRFQLLIDAMNQGPSADPFYDYNYYFSTETTAKVGESAYPNYSRYSNPEVDAALNAVK
GIDSTDAAKRQPYFDVVQTRVEQDMPYIPILTGGTTSEYHSDKFTGWPTKDNLYAFPAVW
SRPDQAQIYKTLKPAGQ

>Contig_New137_39
MKWQRSRFVAAAAASALVLAGCSSTAGSSGDVTLNYWIWDSAQQPGYQKCADAFSKAN
PGIGVKITQYGWDDYWTTLTAGLVSGEGPDVFVSHLNHYPELASQNQIMPIDDVVESKQV
DLSSYRKGLADLWVGQDGKRYGLPKDYDTVAVFANQKMLDDAGITAQQLNTMAWNPR
DGGTFEKTIAHLTVDKKGVRGDQPGFDKANVAVYGLGLNASGGGFGQTEWSQYAFSDG
WTHSDKNPWGTRWNYGDPKFLETIGWFQSLAKKGYLPTLQVAGGGKVGRQPDNYGAGK
YAMVTEGSWNTKTYFQMKGVKTTIAPVPAGPNGQRASMFNGLADNIAAGTRHPDEAKKL
VAFLGSKACQDLTAAEGVAFPAVESSAQVSKDAFAKQGIDTASFQVPIDENSTHLAPVAQH
WTELQAVMNPAMDAIMSLTAEPDSLKAANQQVNALFAQ

>Contig_New142_66
VAGARRVGRQDGLLADPEHTQAAGVTGRSGRGPAVGVEEGRAGGQERGDPDAAGRRDA
GEPRRRGRRGRGGGRRRRRRRCGRFAGAPAQQNQCRAEHGTADYGSVQGFPPISLVKSGS
TIMGMGEEETTAAEPMTETKPLSERDLRVSDDEREHVVGVLQKAIGRGMIDLDEFTERTDR
ALASRTRGELNAVLADLAGLFHPAAAVAAAPAYAPPIGYGGYSPGQRFELNAKYSSLVRS
GPWVVPPEMVVRNKYGSTKLDFTEAQVQSPVVHIELDAKWGSVEVIIPEHAAVDVNSITDI
KFGSLEDKTRSNGRMGNPRFVLTGRVHGGSLVVRHPRRGLFG

>Contig_New153_197
MSLVKATLHTNQGDIHLNLLPDHAPKTVANFVGLAEGTKEYTQPNAAGTNSGPFYDGSIF
HRVIDGFMLQGGDPTGTGRGGPGYKFGDEFHPELQFSKPYLLAMANAGPGTNGSQFFITV
APTTHLNFRHTIFGEVADQESRNVVDTIARTSTGPADRPLTDIVIEKITIEH

>Contig_New153_434
VAKAKFERTKPHVNIGTIGHVDHGKTTLTAAITKVLHDKYPELNESRAFDQIDNAPEEKQR
GITINISHVEYQTEKRHYAHVDAPGHADYIKNMITGAAQMDGAILVVAATDGPMPQTREH
VLLARQVGVPYIVVALNKADMVDDEEILELVELEVRELLSSQEFPGDDAPVVRVSGLKALE
GDEKWAEAVLELMAAVDDSVPDPVRELDKPFLMPIEDVFTITGRGTVVTGRVERGQINVN
EEVEIVGIREKSTKTTVTGVEMFRKLLDSGQAGDNVGLLVRGIKREDVERGQVVVKPGTTT
PHTDFEGRVYILSKDEGGRHTPFFNNYRPQFYFRTTDVTGVVTLPEGTEMVMPGDNTDITV
ALIQPVAMDEGLRFAIREGGRTVGAGQVTKINK

>Contig_New153_467
VTSDNGTSAGHDLTELSDEQVHVALGDEESAHLEPVEVSDSEVDDDASVDDADAAASAD
EADEPAADDEDPVAKLRAELVAAPGEWYVVHSYAGYENKVKTNLETRTQTLDVEDYIFQI
EVPTEEVTEIKNGQRKQVQRKVLPGYILVRMDLNDASWSAVRNTPGVTGFVGATSRPSPL
TVDEVLKFLAPKVESEAPAKAGKGDSTSASSQLGGPTVEVDFEIGESVTVMDGPFATLPATI
SEVNIDGQKLKVLVSIFGRETPVELSFNQVSKI

>Contig_804_20
VALIEQVGAREILDSRGNPTVEVEVALDDGTLARAAVPSGASTGEHEAVELRDGDTGRYN
GKGVERAVAAVLDEIGPEMVGIEAVDQRIVDQKLVDLDGTPAKSRLGANAILGVSLAVAK
AAAESAELELFRYLGGPNAHVLPVPMLNILNGGSHADSNVDVQEFMIAPIGAETFREALRW
GAEVYHSLKSVLKGRGLSTGLGDEGGFAPNLANNREALDLILQAIEKAGYTPGRDVALAL
DVAATEFFADGAYTFEGSKKSAEQMSAYYAELIRDYPMVSIEDPLSEDDWDGWVTLTAEV
GEKVQIVGDDLFVTNPDRLEEGITRRAANALLVKVNQIGTLSETLDAISLATSFGYKSMMS

HRSGETEDTFIADLAVATGVGQIKTGAPARGERIAKYNQLLRIEETLGDAARYAGELAFPRF
SAES

>Contig836_18
MNGRKNCPLSTMTPPSGWLTVSGNTAPIPDADRPRRPTVRIAVPREIKKHEYRVALTPAGV
HELVSRGHDVFVETGAGAGSSITDEEYVDAGAKILATADQTWAEGELVLKVKEPIAEEYPR
LRAGQVLFTYLHIAADRPLTEALLAAGTTAIAYETVQTASGALPLLAPMSEVAGRLAPQVG
AFSLMKPSGGRGVLPGGIPGVHPARVVVIGGGVAGLNAARVALGLGSDVEILDTNVDRLR
QIDNDFGGRIRTVTSNRLSVEESVLQADLVIGAVLVPGAKAPKLVSNDLVARMKPGSVLVD
IAIDQGGCFADSRPTTHDDPTYTVHESLFYCVANMPGAVPRTSTYGLTNVTLPYAVQLAEH
GWKAALQADAALAKGLNTHAGALTNGPVAVAHDLPHTPLDTVLA

>Contig_882_10
VVTIGTVAAGTDCTAGSQLTYTLPMAVAWRPPGDSGGRCRSEGVPAVTDRISHWIDGKPF
TGTAERSGEVFDPATGQVRAHVDFAGDAEVEAAVAAAKAALPGWRGTSLAGRTRVMFA
FRELLSARKHELAKIITSEHGKVESDAAGEIARAIENVEYACGAAQLLKGGFSENASTGVD
VYSIAQPLGVVGVISPFNFPAMVPLWFVPNALACGNTVVLKPSEKDPSAAVFIAELFAEAG
LPAGALNVLHGDKVAVDGLLEHADVKAISFVGSTPIARYVYETGTRYGKRVQALGGAKN
HMVVLPDADLDLAADAAVSAGFGSAGERCMAVSVVVAVDPVGDSLVEKIAERIARLRVG
DGRDPESEMGPLVTAAHHTRVESYVDAGVASGASLVVDGRGIEVSDASDGFWLGPTLFDH
VRPEMSIYTDEIFGPVLAVVRATSYDDALALINANPYGNGTAIFTGDGAAARRFQNEVEVG
MVGVNVPIPVPVGYYSFGGWKDSLFGDSHAYGPEGFHFFTRTKVVTSRWPDRSHAGVNL
GFPRNS

Table 5S. BLAST analysis using UniProt databank of proteins identified by MS analysis.

Query Name	Query Length (AA)	UniProt Acc.	Hit Length (AA)	Hit Description	HSP Length	HSP start	HSP end	Positives	Total Score	Total E-Value
Contig_New11_45	291	C7N033	225	Zn-dependent hydrolase, glyoxylase [Svir_25780] [*Saccharomonospora viridis*]	224	67	291	188/225 (83%)	365	1E-99
Contig_New14_31	307	C6WDI8	297	Putative uncharacterized protein [Amir_5813] [*Actinosynnema mirum*]	292	15	307	199/293 (67%)	278	7E-73
Contig_New14_52	304	C7MZE0	280	Phage shock protein A (IM30) [Svir_25130] [*Saccharomonospora viridis*]	276	26	302	236/278 (84%)	445	1E-123
Contig_New34_11	542	C6WPY2	542	Delta-1-pyrroline-5-carboxylate dehydrogenase [Amir_1082] [*Actinosynnema mirum*]	540	1	541	439/541 (81%)	796	0
Contig_New42_138	660	C7MQM 9	663	Acetyl-coenzyme A synthetase (EC 6.2.1.1) [Svir_36010] [*Saccharomonospora viridis*]	661	1	662	596/663 (89%)	1142	0
Contig_New51_10	540	C7MSN3	538	Glucose-6-phosphate isomerase [Svir_15960] [*Saccharomonospora viridis*]	518	5	523	392/519 (75%)	676	0
Contig_New51_11	373	C6W8G1	370	Transaldolase [Amir_5187] [*Actinosynnema mirum*]	369	4	373	308/370 (83%)	263	3E-69
Contig_New63_2	153	C7MYW 9	151	Peroxiredoxin [Svir_10330] [*Saccharomonospora viridis*]	151	1	152	138/152 (90%)	263	3E-69
Contig_New63_221	614	C6WLU4	519	GMP synthase, large subunit [Amir_6529] [*Actinosynnema mirum*]	515	99	614	462/516 (89%)	864	0
Contig_New63_232	503	C7MU21	514	Inosine-5'-monophosphate dehydrogenase (EC 1.1.1.205) [Svir_04740] [*Saccharomonospora viridis*]	501	2	503	471/502 (93%)	863	0
Contig_New79_28	525	C7N048	495	SSU ribosomal protein S1P [Svir_25930] [*Saccharomonospora viridis*]	483	42	525	422/484 (87%)	789	0
Contig_New80_94	514	Q73WB3	515	LuxE-like Putative uncharacterized protein [MAP_2747] [Mycobacterium paratuberculosis]	499	15	514	356/506 (70%)	567	1E-159
Contig_New81_23	403	A4F6A7	401	Cytochrome P450-like enzyme (EC 1.14.-.-) [*cypA*] [*Saccharopolyspora erythraea*]	395	5	400	313/396 (79%)	531	1E-149
Contig_New89_176	590	Q0SHK8	576	Probable dihydrolipoyllysine-residue succinyltransferase (EC 2.3.1.61) [RHA1_ro01151] [*Rhodococcus* sp.]	589	1	590	402/594 (67%)	630	1E-178
Contig_New89_177	482	C7MYY 5	457	Dihydrolipoyl dehydrogenase (EC 1.8.1.4) [Svir_10500] [*Saccharomonospora viridis*]	455	26	481	411/456 (90%)	754	0
Contig_New89_185	466	C7MYZ1	367	Branched-chain-amino-acid aminotransferase (EC 2.6.1.42)[Svir_10560] [*Saccharomonospora viridis*]	366	100	466	329/367 (89%)	609	1E-172
Contig_New94_58	251	A4F5V3	252	Putative uncharacterized protein [SACE_0074] [*Saccharopolyspora erythraea*]	156	30	186	105/157 (66%)	166	1E-39
Contig_New102_120	332	A0AE48	334	Putative oxidoreductase [SAMR1047] [*Streptomyces ambofaciens* ATCC]	331	1	332	197/333 (59%)	301	5E-80
Contig_New110_24	565	C7MVE4	558	Uncharacterized conserved protein [Svir_27920] [*Saccharomonospora viridis*]	530	35	565	390/534 (73%),	673	0
Contig_New116_115	261	C7MSM 6	261	Triosephosphate isomerase (EC 5.3.1.1) [Svir_15890] [*Saccharomonospora viridis*]	260	1	261	234/261 (89%)	432	1E-119
Contig_New116_116	407	C6W8H2	388	Phosphoglycerate kinase (EC 2.7.2.3) [*pgk*] [*Actinosynnema mirum*]	402	3	405	342/403 (84%)	592	1E-167
Contig_New116_117	475	C7MSM 4	234	Glyceraldehyde-3-phosphate dehydrogenase (EC 1.2.1.12) [Svir_15870] [[*Saccharomonospora viridis*]	326	149	475	292/327 (89%)	553	1E-155
Contig_New120_88	255	C7MXG 2	255	Enoyl-(Acyl-carrier-protein) reductase (NADH) [Svir_22330] [*Saccharomonospora viridis*]	251	3	254	229/252 (90%)	432	1E-119
Contig_New120_141	156	C6W9Y6	150	FHA domain containing protein [Amir_5356] [*Actinosynnema mirum*]	153	1	154	142/154 (92%)	272	1E-71
Contig_New122_10	145	Q82QP6	144	Putative heat shock protein [hsp18_1] [*Streptomyces avermitilis*]	142	1	143	126/143 (88%)	218	2E-55
Contig_New125_37	430	C6WGY 1	430	Aminotransferase class-III [Amir_2104] [*Actinosynnema mirum*]	424	3	427	322/427 (75%)	547	1E-153
Contig_New129_4	311	C7MZK8	300	UDP-glucose pyrophosphorylase [Svir_32670] [*Saccharomonospora viridis*]	297	11	308	214/298 (71%)	372	1E-101
Contig_New129_94	228	C7MZQ7	235	Phosphopantetheinyl transferase [Svir_33220] [*Saccharomonospora viridis*]	226	2	228	162/229 (70%)	255	2E-66
Contig_New129_97	542	C7MZR0	536	Acyl-CoA synthetase (AMP-forming)/AMP-acid ligase II [Svir_33250] [*Saccharomonospora viridis*]	534	5	539	487/535 (91%)	894	0
Contig_New129_237	364	D2PYI5	360	ATPase associated with various cellular activities AAA_5 [*Kribbella flavida*]	327	37	364	283/328 (86%)	492	1E-137

Contig_New133_66	84	C7MR76	67	Cold-shock DNA-binding protein family [Svir_37160] [*Saccharomonospora viridis*]	66	18	84	67/67 (100%)	163	3E-39
Contig_New135_138	584	C6WKL4	583	Succinate dehydrogenase or fumarate reductase, flavoprotein subunit (EC 1.3.99.1) [Amir_6464] [*Actinosynnema mirum*]	583	1	584	539/584 (92%)	1025	0
Contig_New135_68	504	C6WKF9	504	Glycerol kinase [Amir_6409] [*Actinosynnema mirum*]	503	1	504	452/504 (89%)	870	0
Contig_New137_25	560	C6WHP4	560	Extracellular solute-binding protein family 5 precursor [Amir_6188] [*Actinosynnema mirum*]	534	26	560	429/536 (80%)	736	0
Contig_New137_39	449	A1R9Z5	446	Putative extracellular sugar-binding protein [AAur_3361] [*Arthrobacter aurescens*]	426	21	447	293/427 (68%)	475	1E-132
Conitg_New138_198	327	A4F798	261	Phenazine biosynthesis PhzC/PhzF protein [SACE_0577] [*Saccharopolyspora erythraea*]	248	77	325	174/259 (67%)	272	4E-71
Contig_New142_66	342	C7MS38	219	Putative uncharacterized protein [Svir_39520] [*Saccharomonospora viridis*]	198	143	341	134/201 (66%)	202	6E-50
Contig_New153_197	172	C7MRQ4	182	Peptidyl-prolyl cis-trans isomerase (EC 5.2.1.8) [Svir_00260] [*Saccharomonospora viridis*]	167	4	171	141/168 (83%)	272	8E-72
Contig_New153_434	397	C7MTK3	397	Elongation factor Tu (EF-Tu) [*tuf*] [*Saccharomonospora viridis*]	396	1	397	353/397 (88%)	667	0
Contig_New153_467	274	C7MTH0	267	Transcription antitermination protein *nusG* [Svir_03630] [*Saccharomonospora viridis*]	173	1	174	206/274 (75%)	345	3E-93
Contig_New153_535	443	C7MTD0	460	NADH dehydrogenase subunit D (EC 1.6.99.3) [Svir_03190] [*Saccharomonospora viridis*]	430	13	443	373/431 (86%)	711	0
Contig804_20	428	C6WJK2	428	Enolase (EC 4.2.1.11) [Amir_0671] [*Actinosynnema mirum*]	426	1	427	386/427 (90%)	703	0
Contig836_18	409	Q8RME8	371	Alanine dehydrogenase [ald] [*Amycolatopsis mediterranei*]	370	39	409	305/371 (82%)	576	1E-162
Contig882_10	544	C6WGY 2	513	Methylmalonate-semialdehyde dehydrogenase [Amir_2105] [Actinosynnema mirum]	494	50	544	367/495 (74%)	646	0

Table 6S. *Amycolatopsis balhimycina* DSM5908 genes arranged in putative operons.

Protein	Gene acronym	Main metabolic pathway/Cellular function (category)[1]	Sequence source[2]
triosephosphate isomerase	TPI	Glycolysis/Gluconeogenesis	Contig_New116_115
phosphoglycerate kinase#	Pgk	Glycolysis/Gluconeogenesis	Contig_New116_116
glyceraldehyde 3-phosphate dehydrogenase	GAPD	Glycolysis/Gluconeogenesis	Contig_New116_117
NADH dehydrogenase I chain E	NuoE	Oxidative phosphorylation	Contig_New153_534
NADH dehydrogenase I chain D	NuoD	Oxidative phosphorylation	Contig_New153_535
glucose-6-phosphate isomerase	PgiA	Glycolysis/Gluconeogenesis	Contig_New51_10
transaldolase	TrA	Pentose phosphate pathway	Contig_New51_11
transketolase	TrK	Pentose phosphate pathway	Contig_New51_12
succinyl-CoA ligase alpha subunit	A-SCS	TCA cycle	Contig_New63_202
succinyl-CoA ligase beta chain	B-SCS	TCA cycle	Contig_New63_203
dihydrolipoamide acyltransferase	SucB	TCA cycle	Contig_New89_176
dihydrolipoamide dehydrogenase	LpdA2	TCA cycle	Contig_New89_177

Table 7S. Upstream regions of selected *A. balhimycina* genes. In bold are shown the PHO box directed repeats (DR) identified by ClustalW and BLAST bl2seq analysis using *S. coelicolor* PHO box DR in the upstream regions of *pstS*.

>PHO box DR in the upstream region of *S. coelicolor pstS* [16]
gttcacccggc gttcatttacg

>New140_38: *pstS*
gttcactttgc gttcatctgga *-50* caggggaac ccgtccatcg gcgctgccta atgtccggat ccggcgcggc *-1*

>New11_4: *phoD*
gttcacccgcg cgcaaaccggc *-50* gttggccg cggcgggata gaaaccgcgc atgaccgaac cgacccattc *-1*

>Contig_New68_128: *ppk*
gttcatctgac cttcaccacgg *-40* cctgcgcaa tccggcgtgc cttagtgaag aatgggggcc *-1*

>446_4: malate dehydrogenase (*mdh*)
gttcaccccct gaccgtttcga ggcgtcaccgc *-90* gagagtgac tggacgcacc cgaacagcga cgcccaggag ggataggctc caagtcacta gccaaagtcg tctcaagctg gagtaccgca *-1*

>New89_177: dihydrolipoamide dehydrogenase (*lpdA2*)
gttcgtcttcg gttccacatag *-145* gctt cgacgccgcg ccgggaaccc gcccactcgt cgagatggcg ggtgtcctcg gagctggctt tccgcagcgt accggcccgt tgcccaccct tggcccgcct gcgcagcgag tcgaagagac ccacctcgac caccctccct cacgagactt ccacccctca ttatcccggt gtccgcgtgt ggtcggcgtc gcacacccgg ctagctcgca gcctcgtccg gcgtagtgac aagatggcga *-1*

>New63_203: *b-scs*
gttcacccgcc tcgacccggcc gccgtcgcg *-191* ctcgggcgct tcctgcgtga cgaaggcctc gaagtcatcc acgccggacc cctcggcacc gttgaacaga tccttcgcac cgccgaacaa gaggacccgg acgcggtcgt agtcctcggt gacgagccgc cggacctgcc cggtctccgt gtcttcaccg acccggaaga agccgcggag tgggcttccg *-1*

>New18_16 *hmaS*
gttcaccgggc gctgacccgcg *-160* cgcggtgcccc ggtgagggtgc ggctgccccgc gcccattttgt ccact gtggactccg gcgcccgccg cggcgggtgt caagctgaca ccgttgatgc ggaattggct tggagccatc ctggggaatg agcgttacac ctatttgacg gaggaatgtc *-1*

>New125_37: *gabT1*
gttgaagcgct gttcacggcat *-39* ccccatac tccttggaga ctcgcagagg aggcgtccga *-1*

>new151_106: *p-pfk*
ggtcagcgcag gttcacgccgc *-182* cgtgcccaccc ggggccggct tcgtaaacca gaaatagcgc tgcacgggct caacgtagca cagggtcacc ggggaccgat ccggcaggtg gtacggcctg caccgatcaa gacgggagcc gctacgctgg cggacgacga aatgtgactg tcatctcccg agaacagacg gaggcttctc *-1*

Table 8S. EMBOSS-GUI Matcher analysis of PHO box DR performed by using *S. coelicolor* PHO box DR in the upstream regions of *pstS*.

**Sequence (gene)**	**Matching analysis Vs gttcacccggc**	**Matching analysis Vs gttcatttacg**
**gttcactttgc (*pstS*)**	Identity: 6/6; Gaps:0/6; Score: 30	Identity: 7/8; Gaps:0/8; Score: 31
**gttcatctgga (*pstS*)**	Identity: 8/10; Gaps:0/10; Score: 32	Identity: 7/8; Gaps:0/8; Score: 31
**gttcacccgcg (*phoD*)**	Identity: 9/9; Gaps:0/9; Score: 45	Identity: 5/5; Gaps:0/5; Score: 25
**cgcaaaccggc (*phoD*)**	Identity: 6/7; Gaps:0/7; Score: 26	Identity: 2/2; Gaps:0/2; Score: 10
**gttcatctgac (*ppk*)**	Identity: 8/11; Gaps:0/11; Score: 28	Identity: 7/8; Gaps:0/8; Score: 31
**cttcaccacgg (*ppk*)**	Identity: 6/6; Gaps:0/6; Score: 30	Identity: 4/4; Gaps:0/4; Score: 20
**gttcacccccct (*mdh*)**	Identity: 8/8; Gaps:0/8; Score: 40	Identity: 5/5; Gaps:0/5; Score: 25
**gaccgtttcga (*mdh*)**	Identity: 3/3; Gaps:0/3; Score: 15	Identity: 4/5; Gaps:0/5; Score: 16
**ggcgtcaccgc (*mdh*)**	Identity: 5/5; Gaps:0/5; Score: 25	Identity: 0/3; Gaps:0/3; Score: 15
**gttcgtcttcg (*lpdA2*)**	Identity: 4/4; Gaps:0/4; Score: 20	Identity: 8/11; Gaps:0/11; Score: 28
**gttccacatag (*lpdA2*)**	Identity: 4/4; Gaps:0/4; Score: 20	Identity: 4/4; Gaps:0/4; Score: 20
**gttcacccgcc (*b-scs*)**	Identity: 10/11; Gaps:0/11; Score: 46	Identity: 5/5; Gaps:0/5; Score: 25
**tcgacccggcc (*b-scs*)**	Identity: 7/7; Gaps:0/7; Score: 35	Identity: 2/2; Gaps:0/2; Score: 10
**gttcaccgggc (*hmaS*)**	Identity: 10/11; Gaps:0/11; Score: 46	Identity: 5/5; Gaps:0/5; Score: 25
**gctgacccgcg (*hmaS*)**	Identity: 7/9; Gaps:0/9; Score: 27	Identity: 3/4; Gaps:0/4; Score: 11
**gttgaagcgct (*gabT1*)**	Identity: 6/9; Gaps:0/9; Score: 18	Identity: 4/5; Gaps:0/5; Score: 16
**gttcacggcat (*gabT1*)**	Identity: 6/6; Gaps:0/6; Score: 30	Identity: 5/5; Gaps:0/5; Score: 25
**ggtcagcgcag (*p-pfk*)**	Identity: 5/7; Gaps:0/7; Score: 17	Identity: 4/5; Gaps:0/5; Score: 16
**gttcacgccgc (*p-pfk*)**	Identity: 9/11; Gaps:0/11; Score: 37	Identity: 5/7; Gaps:0/7; Score: 17

Table 9S. Composition of fermentation media used.

Compound	LP medium	LG medium
KH_2PO_4	0,6 mM	1,8 mM
$(NH_4)_2SO_4$	24 mM	24 mM
NaCl	1 g/l	1 g/l
$MgSO_4.7H_2O$	1.5 g/l	1.5 g/l
$FeSO_4.7H_2O$	0.02 g/l	0.02 g/l
Na_3-citrate.$2H_2O$	0.05 g/l	0.05 g/l
$ZnSO_4$.7H2O	0.02 g/l	0.02 g/l
$MnSO_4.H_2O$	0.01 g/l	0.01 g/l
$CaCl_2.2H_2O$	0.01 g/l	0.01 g/l
Antifoam	0.1 ml/l	0.1 ml/l
Vitamin solution*	1x	1x
Glucose	12 g/l	6 g/l

* Vitamin solution contained (per liter): 0.05 g biotin, 1 g Ca-pantothenate, 1 g nicotinic acid, 25 g myo-inositol, 1 g thiamine-HCl, 1 g pyridoxine-HCl and 0.2 g para-aminobenzoic acid.

Tab. 10S. Primers used for qRT-PCR experiments.

Gene	Forward (5'-3')	Reverse (5'-3')	Predicted size (bp)
phoP	acgtcaccaagccgtactcg	Gagcaggtcgaactccttga	200
phoR	cgtcgccgaactctacgaac	Gcgaaaggtcgatttccatc	200
phoD	tctacgcccaatgggatgac	Gtagcggaagctgcggtaga	188
pstS	acaagctctccggtgtggac	Tctggaagttgtcggtggtg	192
ppk	cacccgttcccctacatctc	Agatgcgccgagatgagttc	191
bbr	agcgatccgggtctgtctc	Ggtcgctcttgagcctttcc	220
vanS	cagcagcaggaagaacagca	Gcctcaaactcaccctcagc	197
dpgA	ctacgactgggaccgtgacc	Gaggttgacgacgacggagt	193
hmaS	tgacttccgattcgactgtcc	Tcggtgaggaccagcttgat	184
bpsD	acgtcgtttcgttcgagcac	Gaagccccggatgtcacag	199
bpsA	ccgcagatcgggttcaacta	Cattccagcatgagcgtcag	188
oxyA	gcacggaaatacaccggaga	Agcttggcgctgaacttgtc	181
bgtfA	gcgaacctcttcacctacgg	Ctgccgaaacccacgtacac	194
hrdb	cctggtggaacaggtgaaca	cctcggagtcctcgatgaag	218
